# Automated analysis of immunosequencing datasets reveals novel immunoglobulin D genes across diverse species

**DOI:** 10.1371/journal.pcbi.1007837

**Published:** 2020-04-27

**Authors:** Vinnu Bhardwaj, Massimo Franceschetti, Ramesh Rao, Pavel A. Pevzner, Yana Safonova

**Affiliations:** 1 Electrical and Computer Engineering Department, University of California San Diego, San Diego, California, United States of America; 2 Qualcomm Institute, University of California San Diego, San Diego, California, United States of America; 3 Computer Science and Engineering Department, University of California San Diego, San Diego, California, United States of America; 4 Center for Information Theory and Applications, University of California San Diego, San Diego, California, United States of America; University of Ottawa, CANADA

## Abstract

Immunoglobulin genes are formed through V(D)J recombination, which joins the variable (V), diversity (D), and joining (J) germline genes. Since variations in germline genes have been linked to various diseases, personalized immunogenomics focuses on finding alleles of germline genes across various patients. Although reconstruction of V and J genes is a well-studied problem, the more challenging task of reconstructing D genes remained open until the IgScout algorithm was developed in 2019. In this work, we address limitations of IgScout by developing a probabilistic MINING-D algorithm for D gene reconstruction, apply it to hundreds of immunosequencing datasets from multiple species, and validate the newly inferred D genes by analyzing diverse whole genome sequencing datasets and haplotyping heterozygous V genes.

## Author summary

Antibodies provide specific binding to an enormous range of antigens and represent a key component of the adaptive immune system. Immunosequencing has emerged as a method of choice for generating millions of reads that sample antibody repertoires and provides insights into monitoring immune response to disease and vaccination. Most of the previous immunogenomics studies rely on the reference germline genes in the immunoglobulin locus rather than the germline genes in a *specific patient*. This approach is deficient since the set of known germline genes is incomplete (particularly for non-European humans and non-human species) and contains alleles that resulted from sequencing and annotation errors. The problem of *de novo* inference of *diversity* (D) genes from immunosequencing data remained open until the IgScout algorithm was developed in 2019. We address limitations of IgScout by developing a probabilistic MINING-D algorithm for D gene reconstruction and infer multiple D genes across multiple species that are not present in standard databases.

## Introduction

Antibodies provide specific binding to an enormous range of antigens and represent a key component of the adaptive immune system [1]. The *antibody repertoire* is generated by *somatic recombination* of the V (*variable*), D (*diversity*), and J (*joining*) germline genes by a process known as V(D)J recombination. During this process, the germline V, D, and J genes are randomly selected, and the gene ends are randomly trimmed and joined together along with some random insertions between the trimmed genes, leading to a huge number of unique recombined sequences. The specificity of an antibody is largely defined by the recombination site referred to as the *third complementarity determining region* (*CDR3*) [2].

Immunosequencing helps in monitoring immune response to disease and vaccination by generating millions of reads that sample antibody repertoires [3]. Information about all germline immunoglobulin genes specific to the *individual* is a prerequisite for analyzing immunosequencing (*Rep-Seq*) data. However, most previous immunogenomics studies have relied on the *population-level* germline genes. As the set of known germline genes is incomplete (particularly for non-Europeans) and contains alleles that resulted from sequencing and annotation errors [4, 5], studies based on population-level germline genes can lead to incorrect results. Moreover, it is difficult to find which known allele(s) is present in a specific individual since the widespread practice of aligning each read to its closest germline gene results in high error rates [5]. Using population-level germline genes rather than individual germline genes can thus make it difficult to analyze *somatic hypermutations* (*SHM*) and clonal development of antibody repertoires [6–8].

Identifying individual germline genes (i.e., *personalized immunogenomics*) is important since variations in germline genes have been linked to various diseases [9], differential response to infection, vaccination, and drugs [10, 11], aging [12], and disease susceptibility [9, 13, 14]. There still exist unknown human allelic variants and the International ImMunoGeneTics (IMGT) database [15] is incomplete even for well-studied human germline genes [16]. The germline genes for less studied albeit immunologically important model organisms remain largely unknown [17, 18]. Assembling the highly repetitive immunoglobulin loci from whole genome sequencing data is difficult [19] and efforts such as the 1000 Genomes Project have led to only limited progress towards inferring the population-wide census of germline immunoglobulin genes [19–21].

Although the personalized immunogenomics approach was first proposed by [22], the manual analysis in this study did not result in a software tool for inferring germline genes. Gadala-Maria et al. [23] developed the TIgGER algorithm for inferring germline genes and used it to discover novel alleles of V genes. The challenge of *de novo* reconstruction of V and J genes was further addressed by Corcoran et al. [24], Zhang et al. [25], Ralph and Matsen [5], and Gadala-Maria et. al. [26]. However, as Ralph and Matsen [5] commented, the more challenging task of *de novo* reconstruction of D genes remained elusive.

The sequences encoded by D genes play important roles in B cell development, antigen binding site diversity, and antibody production [27]. Safonova and Pevzner [28] recently developed the IgScout algorithm for *de novo* inference of D genes using immunosequencing data. Unlike algorithms for de novo inference of V and J genes [23, 24], it does not rely on alignments against closest germline genes that might lead to erroneous inferences [29, 30]. Instead, IgScout uses the observation that the most abundant *k*-mers in CDR3s arise from D genes (a *k-mer* refers to a string of length *k*). However, IgScout lacks a probabilistic model and has limitations with respect to inferring short D genes and D genes that share substrings with other D genes. It relies on the knowledge of *k* such that each *k*-mer occurs in a single D gene (information that is often unavailable) and uses those *k-*mers as *seeds* in its *seed extension* procedure. However, if a *k*-mer seed occurs in multiple D genes, IgScout might miss some D genes altogether and sometimes even produce inaccurate results. To bypass this problem, IgScout attempts to select large *k* to guarantee that each *k*-mer occurs in a single D gene (e.g., *k* = 15 for human D genes). However, using long *k*-mers as seeds results in missing D genes that are shorter than those *k-*mers. Thus, for species with limited information about the range of D gene lengths, IgScout is bound to make errors.

Our MINING-D algorithm uses a probabilistic model and addresses above limitations of IgScout. We applied MINING-D to nearly 600 publicly available Rep-seq datasets from humans, mice, camels, rhesus macaques, rats, and rabbits. In total, MINING-D inferred 13, 6, 4, 8, 12, and 15 novel D genes using human, mouse, rat, macaque, camel, and rabbit datasets, respectively. We validated 25 out of these 58 novel D genes—2, 1, 3, 8, 8, 3 D genes for human, mouse, rat, macaque, camel, and rabbit datasets, respectively—using Whole Genome Sequencing data. We further analyzed the usage of D genes in diverse Rep-seq datasets to analyze potential associations between the usage of a D gene and an environment, *i*.*e*., a health condition, a tissue, or a cell type.

## Methods

### Probabilistic model of CDR3 generation

**Fig 1:**
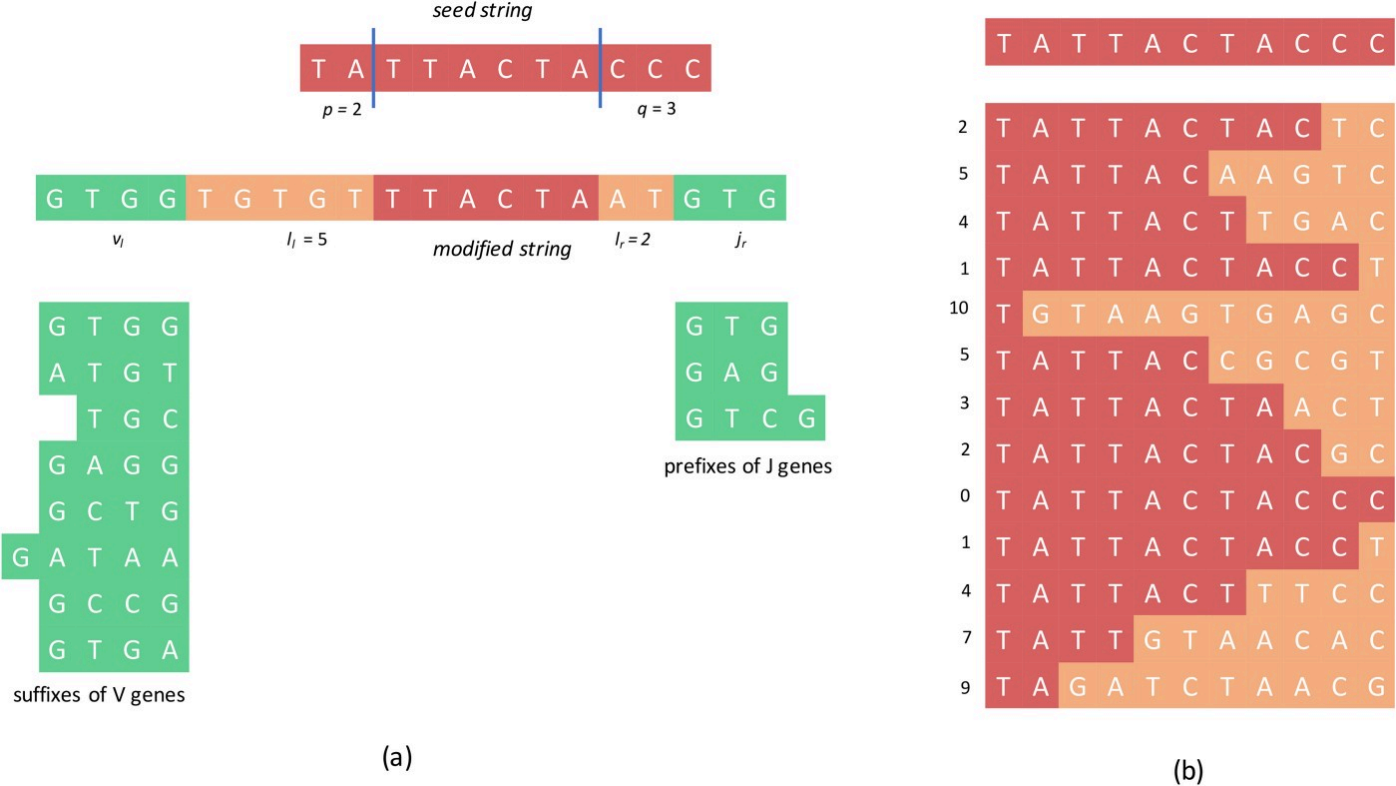
**Transformation of a seed string representing a D gene into a modified string representing a CDR3 (a) and a set of modified strings generated according to a simple probabilistic model (b)**. (a) The symbols in red, yellow, and green in the modified string denote the symbols from the truncated seed string, random insertions, and V suffixes/J prefixes, respectively. The sets of V suffixes and J prefixes are shown below the modified string. Note that the sequences shown here are only for illustration and do not correspond to any real genes. (b) In a simple probabilistic model, suffixes of length *k* are trimmed from the seed string, and the trimmed string is extended by *k* random symbols, where *k* (shown by numbers on the left) is chosen uniformly at random. Note that in most cases, there are multiple ways a modified string can be generated from the original string. For example, the first modified string can be generated from the original string by trimming the suffix “CC” and adding the string “TC” or by trimming the suffix “CCC” and adding the string “CTC”.

The transformation of a D gene (a *seed string*) to a CDR3 (a *modified string)* can be modeled by the following probabilistic model. The seed string *s* is *trimmed* at two randomly chosen locations *p* and *q* (*p+q* ≤ *|s|*, where |.| denotes the length of a string) such that the first *p* and the last *q* symbols of *s* are removed (Fig 1(A)). The resulting string is extended on the left and on the right by randomly generated strings *e*_*l*_ and *e*_*r*_ of randomly selected lengths *l*_*l*_ and *l*_*r*_ respectively. The resulting string is further extended on the left by a randomly chosen string *v*_*l*_ from a set of strings *V*_*cdr3*_ and on the right by a randomly chosen string *j*_*r*_ from a set of strings *J*_*cdr3*_ to form a *modified string c*.

The seed string *s* in the above model corresponds to a D gene, the strings *e*_*l*_ and *e*_*r*_ correspond to the random insertions, and *V*_*cdr3*_ and *J*_*cdr3*_ correspond to the sets of suffixes of V genes and prefixes of J genes that form parts of the CDR3 sequences. All random variables in the model are drawn according to a joint distribution on all the variables.

### D gene inference and the trace reconstruction problem

Given a set *C* = {*c*_*1*_, *c*_*2*_, *c*_*3*_, …, *c*_*N*_} of independently generated instances of the modified strings generated from an unknown set of seed strings *S* = {*s*_*1*_, *s*_*2*_, …, *s*_*M*_}, the D genes inference problem is to reconstruct the set *S* of seed strings. This problem can be thought of as a version of the *trace reconstruction problem* in information theory [31] *i*.*e*., reconstruction of an unknown string *s* given a collection of its *traces* generated according to a given probabilistic model. In the trace reconstruction problem, an unknown string *s* yields a collection of traces, each trace independently obtained from *s* by deleting each symbol with a given probability. In the D genes inference problems, traces are generated according to a more complex probabilistic model with multiple parameters.

### A simple probabilistic model

Although the described variant of the trace reconstruction problem represents an adequate probabilistic model for the VDJ recombination, estimating a joint distribution on the variables that accurately mimics the real recombination events is a difficult task. For the sake of simplicity and to develop an intuition for the MINING-D algorithm, we consider a simpler probabilistic model that is based on a single seed string *s* (representing a single D gene) rather than a set of strings (representing multiple D genes) that gets trimmed only on one side (Fig 1(B)).

Let *s* be a seed string in an alphabet A. The seed string generates a modified string *c* according to the following probabilistic process:

1. A *trimming integer k* is sampled uniformly at random from [0, *|s|*], and the suffix of *s* of length *k* is trimmed.
2. The resulting string is extended by *k* symbols on the right where each symbol is uniformly selected at random from the alphabet A.

Note that a seed string may generate the same modified string for different values of the trimming integer *k*. For example, a seed string ATGA may generate a modified string ATCC for *k* = 2 (with probability 1/5*1/16 in the case of 4-letter alphabet A), or a modified string ATCC for *k* = 3 (with probability 1/5*1/64), or a modified string ATCC for *k* = 4 (with probability 1/5*1/256). The probability *P*(*c*|*s*) that a seed string *s* generates a modified string *c* depends only on the length *m* of their longest shared prefix and is given by

$$P(c|s)=\frac{1}{|s|+1}\sum_{k=0}^{m}\frac{1}{{\left|A\right|}^{|s|-k}}$$ (1)

$$=\frac{1}{(|s|+1){\left|A\right|}^{\left|s\right|}}\sum_{k=0}^{m}{\left|A\right|}^{k}$$ (2)

$$=\frac{1}{(|s|+1){\left|A\right|}^{\left|s\right|}}\times \frac{{\left|A\right|}^{m+1}-1}{|A|-1}$$ (3)

$$=K(\left|s|,|A\right|)\times ({\left|A\right|}^{m+1}-1)$$ (4)

where K(|s|,|A|) is a constant given length of the seed string and the alphabet size. Given a set *C* = {*c*_*1*_, *c*_*2*_, *c*_*3*_, *…*, *c*_*N*_} of *N* modified strings independently generated from the same seed string *s*, the probability that *s* generates *C* is computed as

$$P(C|s)=\prod_{i=1}^{N}P(c_{i}|s)$$ (5)

### String reconstruction problem

Given a set of modified strings *C* generated by an unknown seed string, find a string *s* maximizing *P*(*C*|*s*)

Maximizing *P*(*C*|*s*) is equivalent to maximizing $\prod_{i=1}^{N}\;K(\left|s|,|A\right|)\times ({\left|A\right|}^{m_{i}+1}-1),$ where *m*_*i*_ stands for the length of the longest shared prefix of *s* and *c*_*i*_. Since K(|s|,|A|) is a constant, it is equivalent to finding a string *s* that maximizes:

$$\mathrm{score}(C|s)=\sum_{i=1}^{N}\log\left({\left|A\right|}^{m_{i}+1}-1\right)$$ (6)

Interestingly, if one ignores the “-1” term above, this problem is equivalent to finding a string *s* that maximizes $\sum_{i=1}^{N}m_{i}$, the number of “red” cells in the matrix shown in Fig 1(B). Given a string *s*, score(*C*|*s*) can be computed in O(|s|*N) time.

### Greedy algorithm for D gene inference

Although the objective function in (6) can be efficiently maximized (see Supplemental Note: Exact Algorithm for solving the String Reconstruction Problem), it is unclear how to generalize that algorithm for the more complex model with multiple D genes and varying lengths of modified strings. We thus describe a suboptimal greedy algorithm that is easier to extend to cases where the assumptions of the simpler model do not hold. The algorithm starts with an empty string and at step *j* extends it on the right by the most abundant symbol in *C* at position *j* and discards from *C* the strings that have symbols that are not the most abundant symbols at position *j* (more details in Supplemental Note: Greedy Algorithm). This procedure is repeated until the length of the resulting string is equal to the length of the seed string *s*.

To account for the complexities of the VDJ recombination process, we need to modify the greedy algorithm described above. Therefore, for the original D gene inference problem from CDR3 sequences, we describe a heuristic algorithm MINING-D (**M**ethod for **IN**ference of **I**mmu**N**oglobulin **G**enes—**D**) inspired by the above greedy algorithm and considering the complexities of real CDR3s.

### MINING-D algorithm

**Fig 2:**
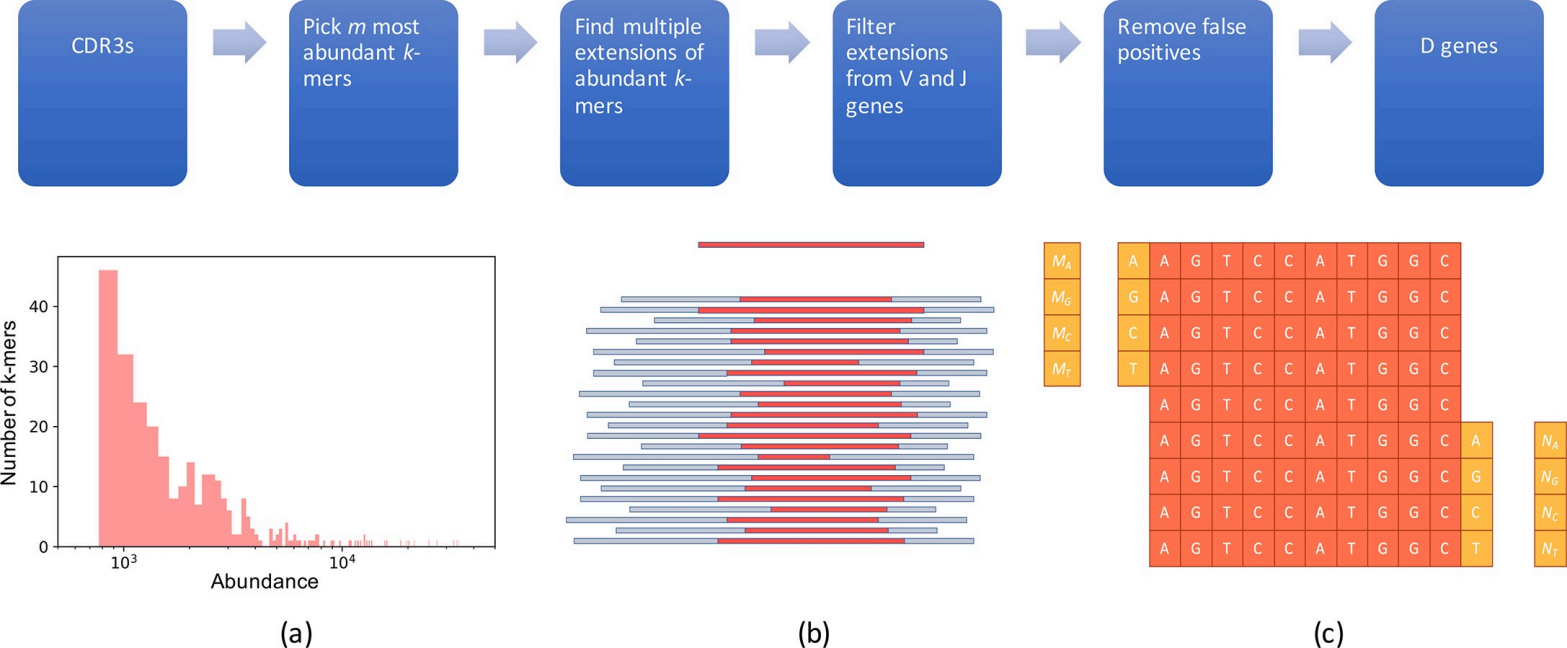
Outline of the MINING-D algorithm. (Top) MINING-D pipeline. (Bottom, a) Abundances of the 300 most abundant 10-mers in the MOUSE dataset vary from 770 to 34,451. (Bottom, b) A D gene (top) and its truncated substrings in various CDR3s of varying lengths (bottom). The red part of a CDR3 is the “surviving” substring of the D gene shown at the top whereas the blue part represents the non-D gene part (parts of V and J genes and random insertions). Some substrings of the original D gene, mostly central, are highly abundant. (Bottom, c) A *k*-mer is extended based on the relative abundances of the four shown (*k*+1)-mers on the left and the four shown (*k*+1)-mers on the right.

Fig 2 presents the outline of the MINING-D algorithm. Although D genes typically get truncated on both sides during the VDJ recombination process, their *truncated substrings* are often present in the newly recombined genes, and, hence, the CDR3s. Therefore, the truncated substrings of D genes are expected to be highly abundant in a CDR3 dataset (Fig 2). MINING-D first finds highly abundant *k*-mers in a CDR3 dataset and then iteratively extends them on both sides to recover the entire D gene based on the elevated relative abundances of the extended substrings. We illustrate the steps of the MINING-D algorithm on a CDR3 dataset constructed from the ERR1759678 sample (MOUSE dataset). The MOUSE dataset corresponds to a pet shop mouse (the strain is unknown) and consists of 124,121 distinct CDR3s. In general, MINING-D does not rely on the productivity of the input sequences and can work on any fragments of the VDJ region (both in-frame and out-of-frame) that cover the entire D gene (as well as short segments of V and J genes).

### Seed selection

MINING-D starts with the *m* most abundant *k*-mers in the CDR3 dataset referred to as *seeds* (default *k* = 10, the selection of the default value of *m* depends upon the species and is described in Supplemental Note: MINING-D Parameters). Most seeds represent substrings of D genes, or strings that have suffixes of V genes or prefixes of J genes as substrings. Our goal is to extend seeds originating from D genes into full-length D genes and filter out seeds originating from (potentially unknown) V and J genes. The abundances of the *m* = 300 (default value for mice datasets) most abundant 10-mers in the MOUSE dataset ranged from 770 to 34,451 (Fig 2).

### Extending seed *k-*mers and the stopping rule

Given a string of length *l*, MINING-D analyzes all its possible extensions on the left and right by a single nucleotide. We test a hypothesis that this string represents the first (last) *l*-mer in some D genes, and thus any nucleotide present immediately on the left (right) of this *l*-mer in CDR3 sequences is a random insertion. If the (*l*+1)-mer resulting by adding the corresponding nucleotide on the left (right) is also a substring of the same D gene, the hypothesis will most likely be rejected, and an extension is made using the most abundant extension symbol.

We start the above procedure with seed *k-*mers. For a highly abundant seed *k*-mer, let the abundances of the four possible extension (*k*+1)-mers on the right be *N*_*A*_, *N*_*G*_, *N*_*C*_, and *N*_*T*_ (Fig 2). We assume a probabilistic model in which a random nucleotide is added to the last *k-*mer according to some distribution. The statistic *S*, where

$$S=\sum_{i\in \{A,G,C,T\}}\frac{{\left(N_{i}-E_{i}\right)}^{2}}{E_{i}}$$

and *E*_*i*_ is the expected abundance under the distribution of the (*k*+1)-mer with the nucleotide *i* added to the right of the *k*-mer is approximately Chi-square distributed with 3 degrees of freedom. We test the null hypothesis that the random nucleotide was added according to a uniform distribution, and, thus, the expected abundances are equal under the null hypothesis. The null hypothesis is accepted or rejected based on the *p*-value of the test. The robustness of the choice of equal abundances of the four (*k*+1)-mers under the null hypothesis can be, to some extent, controlled by choosing a significance threshold to which the *p*-value is compared to accept or reject the hypothesis. Having a low significance threshold will lead to rejection of the hypothesis only when the observed distribution of the abundances of the four (*k*+1)-mers is very different from the uniform distribution, most likely in the case where one of the (*k*+1)-mers is much more abundant than the others (see also Supplemental Note: MINING-D Parameters). The statistical test is run on both the distributions–one with the abundances of the four (*k*+1)-mers corresponding to the extensions on the left and the other corresponding to the extensions on the right. If one of the two hypotheses is rejected, the *k*-mer is extended to the most abundant (*k*+1)-mer corresponding to the rejected hypothesis. If both the hypotheses are rejected, the extension is made corresponding to the hypothesis with a lower *p*-value of the test. In any case, if the *k*-mer is extended to a (*k*+1)-mer, the procedure is repeated until both the hypotheses are accepted. Thus, for every highly abundant seed *k*-mer, we generate a string containing this *k*-mer.

### Finding multiple extensions of seed *k*-mers

**Fig 3:**
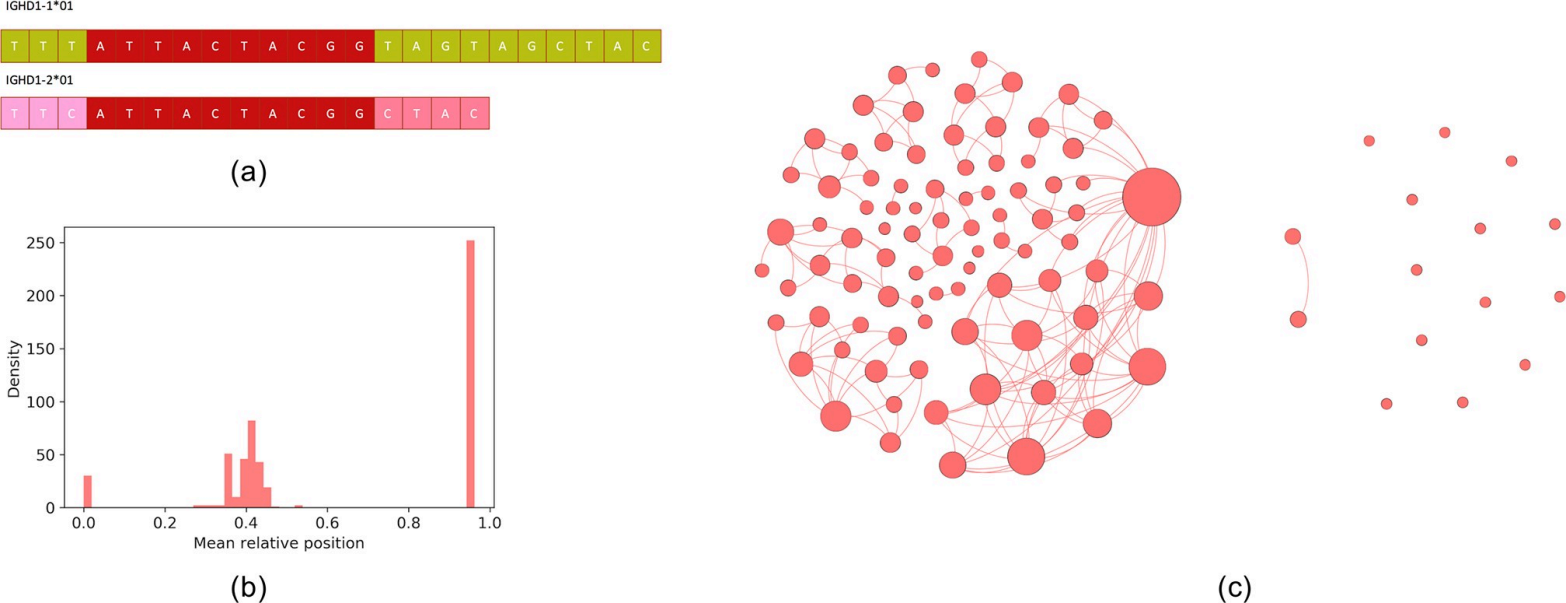
Details of MINING-D algorithm. (a) The 10-mer ATTACTACGG is present in two mouse D genes. (b) The mean relative positions of the extensions in the MOUSE dataset. The relative positions of the extensions form three clusters each corresponding to one of the V, D, and J gene segments. (c) Similarity graph on all extensions corresponding to D genes before filtering extensions or clique merging for the MOUSE dataset (left) and after filtering extensions and merging cliques (right). The size of a node represents its degree.

Some highly abundant *k*-mers can be substrings of multiple D genes as shown in Fig 3(A). Following the procedure above, if we start with a *k*-mer that is a substring of multiple D genes, its extension will most likely correspond to the more abundant D gene in the CDR3 dataset (among D genes containing this *k*-mer). Therefore, sometimes multiple extensions are desired from a single abundant *k*-mer. However, since it is not clear how to avoid false positives in the case of multiple extensions, the IgScout algorithm [28] uses long seed *k*-mers (that are unique among all D genes), thus bypassing the multiple extension problem. Although this approach works for species with partially known germline genes, it is unclear how to select *k* for species with unknown germline genes and short germline genes.

To address this limitation of IgScout, we modified the extension procedure described above. After rejecting a hypothesis at any step (say *j* for *j* ≥ *k*) and extending the *j*-mer to the most abundant (*j*+1)-mer, we further test the hypothesis that the remaining three (*j*+1)-mers follow a random uniform distribution. If the *j*-mer was a part of two D genes and the selected (*j*+1)-mer corresponds to the more abundant D gene among those, the abundance of the (*j*+1)-mer corresponding to the lesser abundant D gene will still be greater than the (*j*+1)-mers corresponding to the random insertions. Hence, the hypothesis will be rejected, and in the next step, extensions of both the (*j*+1)-mers are looked for in an independent manner, leading to multiple extensions from a single abundant *k*-mer. On the MOUSE dataset, the 300 most abundant 10-mers lead to 544 extensions.

### Filtering extensions originating from V and J genes

Since the CDR3 sequences contain some suffixes of V genes and prefixes of J genes, many highly abundant *k*-mers in the CDR3 dataset originate from these suffixes/prefixes rather than D genes. Therefore, it is important to classify the extensions as corresponding to V, D, or J genes while trying to infer D genes from CDR3 sequences. This problem becomes challenging when the V and J genes are unknown.

Since parts of the V, D, and J genes appear in order in each CDR3 sequence, we use the mean relative position of an extension in the CDR3 dataset to classify it as corresponding to one of the V, D, or J genes. We define the relative position of a substring *s* in a CDR3 sequence *c* as follows:

$$RP_{c}(s)=\frac{I_{c}(s)}{|c|-|s|+1},$$

where *I*_*c*_(*s*) is the index of the substring *s* in the list of all the substrings of length |*s*| in *c* ordered from first to last. The normalization by the total number of substrings of length |*s*| in *c* is done to compare the relative positions among CDR3s of varied lengths. The relative position of an extension in the entire CDR3 dataset is taken as the mean of the relative positions of the extension in all the CDR3 sequences of which it is a substring. Looking at the relative positions of the extensions of *k*-mers has some advantages over looking at the relative positions of the *k*-mers as explained in the Supplemental Note: Defining Relative Positions. The mean relative positions of the extensions of abundant 10-mers from the MOUSE dataset are shown in Fig 3(B). Since the central cluster most likely corresponds to the extensions corresponding to the D genes, MINING-D discards the extensions in the left and right clusters.

However, not all the unique extensions in the central cluster correspond to different D genes. The extensions are first filtered according to the method described in the Supplemental Note: Removing Unidirectional Extensions. Out of the 544 extensions corresponding to the MOUSE dataset, 123 remained after filtering out *unidirectional* extensions, out of which only 52 were unique. Of these 52, only 19 were in the central cluster.

### Removing false positives

To reduce the number of reconstructions per D gene, we construct an undirected *similarity graph* on the inferred extensions. Two extensions are adjacent in the graph if they are *similar*. The distance between extensions *e*_1_ and *e*_2_ is defined as *Dist*(*e*_1_,*e*_2_) = min (*|e*_1_|, |*e*_2_*|*)–|*substring*(*e*_1_,*e*_2_)|, where *substring*(*e*_1_,*e*_2_) is the longest common substring of *e*_1_ and *e*_2_. It denotes the number of nucleotides, at the edges, of one extension that need to be changed or deleted to transform it to the other extension or a substring of the extension. The larger this number, the more dissimilar the extensions are. We connect extensions *e*_1_ and *e*_2_ with an edge if *Dist*(*e*_1_,*e*_2_) does not exceed a threshold *maxDist* (the default value is 2).

Cliques in the constructed graph correspond to groups of highly similar extensions. For every clique in the graph, we find the longest common substring among the extensions and extend it to form a new string. This new string then replaces all the extensions that formed the clique. After this clique merging procedure, only 15 of the 19 extensions remained in the MOUSE dataset. Fig 3(C) shows the similarity graph among the extensions before and after filtering unidirectional extensions and merging cliques for the MOUSE dataset. To generate a comprehensive database of D genes from multiple datasets corresponding to different individuals of the same species and health condition, inferred D genes from the datasets were put together and processed (substrings were removed and similar D genes were merged).

### Computing usage of the inferred D genes

Given a set of D genes, we say that a *k*-mer is *unique* if it occurs in a single D gene from this set. We limit attention to *k*-mers that are at least *K*_*min*_ nucleotides long (default value *K*_*min*_ = 8) and say that a CDR3 sequence *c* is *formed* by a D gene *d* if c contains a unique *k*-mer from *d* but does not contain unique *t*-mers from other D-genes for *t* ≥ *k*. A CDR3 sequence is *traceable* if it is formed by a D gene and *non-traceable* otherwise. The usage of a D gene is defined as the proportion of the traceable CDR3 sequences that were *formed* by the D gene.

## Results

### Immunosequencing datasets

We analyzed 588 immunosequencing datasets from 14 publicly available NCBI projects:

- **Human**
  1. **Allergy**. 24 peripheral blood mononuclear cell (PBMC) and bone marrow datasets from six allergy patients from the NCBI project PRJEB18926 [32].
  2. **Flu vaccination**.
    - 95 datasets taken at different times after vaccination from the NCBI project PRJNA324093 corresponding to different types of cells from eight individuals [33].
    - 18 PBMC datasets taken either before vaccination or at least two weeks after the vaccination from three individuals from the NCBI project PRJNA349143 [34].
  3. **Healthy**. 28 PBMC datasets corresponding to either IgG or IgM isotypes from three individuals from the NCBI project PRJNA430091 [35].
  4. **Cord Blood**. 6 datasets corresponding to cord blood samples from five individuals from the NCBI project PRJNA393446.
  5. **Intestinal**. 35 datasets from seven individuals corresponding to different types of isotypes and cell types from the tissues ileum mucosa and colon mucosa from the NCBI project PRJNA355402 [36].
  6. **Multiple Sclerosis**. 32 datasets from four multiple sclerosis patients corresponding to various tissues with different stages of the disease from the NCBI project PRJNA248475 [37].
  7. **Hepatitis B**.
    - 142 datasets corresponding to IgG isotype and various cell types from nine individuals following a Hepatitis B primary vaccination from the NCBI project PRJNA308566.
    - 107 datasets corresponding to IgG and IgM isotypes and various cell types from nine individuals following a Hepatitis B booster vaccination from the NCBI project PRJNA308641.
- **Mouse**. 71 datasets from various cell types (pre-B cells, naive B cells, plasma cells) of 20 untreated and antigen-immunized mice from the strain C57BL/6J, and naive cells of four Balb/c mice and three pet mice from the NCBI project PRJEB18631 [38].
- **Macaque**. 7 datasets from three Indian and four Chinese origin rhesus macaques from the NCBI project PRJEB15295 [24].
- **Camel**. 6 datasets corresponding to the VH and VHH isotypes from three camels from the NCBI project PRJNA321369 [39].
- **Rat**. 10 datasets, each corresponding to an immunized rat of Wistar strain from the NCBI project PRJNA386462 [40].
- **Rabbit**. 7 datasets corresponding to spleen and PBMC of three New Zealand rabbits at different stages of a multi-step immunization from the NCBI project PRJNA355270 [41].

Some immunosequencing datasets in a project represent different samples of immunosequencing data from the same environment representing the same individual, tissue, isotype, etc. (e.g., Donor 1, bone marrow sample 1 and Donor 1, bone marrow sample 2). We merged sequences in such datasets to construct a larger CDR3 dataset corresponding to the same environment. Supplemental Note: Immunosequencing Datasets presents summaries of all immunosequencing datasets analyzed in this study. Meta-categories of these datasets were created for different types of analyses and are shown in Table 1.

**Table 1:** Meta-categories of datasets.

Meta-category	Datasets	Condition(s)
Healthy PBMC	Allergy	PBMC Either before vaccination or at least 2 weeks after (flu vaccination)
	Flu Vaccination	
	Healthy	
Healthy PBMC & Bone Marrow (BM)	Allergy	PBMC or Bone Marrow Either before vaccination or at least 2 weeks after (flu vaccination)
	Flu Vaccination	
	Healthy	
Tissue Specific	Intestinal	All datasets
	Cord Blood	
Stimulated Datasets	Flu Vaccination	All datasets
	Hepatitis B	
	Multiple Sclerosis	
Non-human	Mouse	All datasets
	Macaque	
	Camel	
	Rat	
	Rabbit	

### Constructing CDR3 datasets

For each immunosequencing dataset, we computed CDR3s using the DiversityAnalyzer tool [42]. Since DiversityAnalyzer uses the set of known V and J genes to compute CDR3s and since V and J genes for camel and macaque are unknown, we used human V and J genes to construct CDR3s for these species. Since some CDR3s may be affected by sample preparation errors, we grouped CDR3s differing by at most 3 mismatches and constructed a consensus CDR3 for each group as described in [28]. Constructing consensus CDR3s also helps concentrate on only the recombinant diversity (and not SHMs) of immunosequencing datasets by removing CDR3s with SHMs to some extent. We ignored datasets with less than 15,000 consensus CDR3s for the inference of D genes.

### Known D genes

The ImMunoGeneTics (IMGT) database [15] contains information about human, mouse, rat, and rabbit germline D genes. We used the IMGT D genes of crab-eating macaques for rhesus macaque analysis and the IMGT D genes of alpacas for camel analysis. Table 2 provides information about the D genes of all these species.

**Table 2:** Information about the D genes in the IMGT database for various species.

Species	# D genes (allelic variations)	# distinct sequences	range of lengths of D genes
**Human**	27 (7)	32	11–37
**Mouse**	31 (8)	28	10–29
**Rat**	35 (2)	35	10–29
**Rabbit**	14 (0)	10	24–42
**Crab-eating macaque**	40 (0)	35	11–42
**Alpaca**	8 (0)	8	11–34

### Inferred D genes

For inference of human D genes, PBMC datasets from Healthy, PBMC Flu Vaccination datasets taken either before vaccination or at least two weeks after vaccination, and PBMC datasets from Allergy datasets were considered (Healthy Human PBMC datasets, Table 1). This was done so as to not include any disease specific changes in the repertoire for inference of D genes. For all other species, all available datasets were used. All inferred genes from an immunosequencing dataset (or multiple datasets) were classified into the following categories based on the IMGT database:

- *Inferred gene in IMGT*–the inferred gene is either (i) the same as a known D gene or a known variation, or (ii) a substring of a known D gene or a known variation, or (iii) a substring of a known D gene or a known variation extended by at most *extension* extra nucleotides at the start and/or the end of that substring (the default *extension* = 3).
- *Novel variation*–the inferred gene differs from a known D gene in the database with percent identity > 75%.
- *Novel gene*–the inferred gene has percent identity < 75% compared to all known D genes in the database.

Table 3 presents information about the number of inferred D genes from each species and their classification into one of the categories above. To benchmark the performance against IgScout, we compared the results of MINING-D and IgScout on all Allergy datasets from the project PRJEB18926 and many non-human datasets (see Supplemental Note: Benchmarking MINING-D against IgScout). For human datasets, IgScout failed to reconstruct seven D genes from the IMGT database from all the datasets, whereas MINING-D only missed three genes.

**Table 3:** Information about inferred D genes. The number of novel genes and variations validated using genomic data (procedure described later) are shown.

Species #Individuals	IMGT Database	# Inferred genes	# Inferred genes in IMGT	# Novel variations (validated)	# Novel genes (validated)
Healthy Humans 20	Human	38	25	8 (2)	5 (0)
Untreated + Immunized Mice 27	Mouse	24	18	5 (1)	1 (0)
Immunized Wistar Rats 1	Rat	16	12	4 (3)	-
Rhesus Macaques 7	Crab Eating Macaque	25	17	6 (6)	2 (2)
Bactrian Camels 3	Alpaca	13	1	12 (8)	-
Rabbit 3	Rabbit	18	3	13 (3)	2 (0)

### Novel Variations

Among the 38 (*m* = 600) inferred D genes from the Healthy Human PBMC datasets corresponding to 20 individuals, 8 were labeled as novel variations, including four variations of the gene IGHD3-10*01, two variations of the gene IGHD3-22*01, and single variations of the genes IGHD2-2*01 and IGHD3-16*02. Table 4 presents the sequences of the validated (validation procedure described later) novel variations of genes in Human and other datasets. Note that although only the sequence TTATGATTAC**A**TTTGGGGGAGTTATCGTTAT was inferred as a novel variation of the gene IGHD3-16*02 (N_Var (IGHD3-16*02)-0) from immunosequencing data, the full sequence GTATTATGATTAC**A**TTTGGGGGAGTTATCGTTATACC was found in genomic reads (more details in next subsection). Information about all inferred variations (including variations that could not be validated using genomic data) is presented in Supplemental Note: Novel Variations.

**Table 4:** Novel variations of D genes validated using genomic data from human, camel, rhesus macaque, mouse, rat, and rabbit datasets. “Original” refers to the sequence in the IMGT database. In three of the inferred sequences, there is an extra nucleotide at the end that was not found in the genomic reads, e.g., the novel variation inferred from mice datasets TTTATTACTACG**A**T**G**GTAGCTACg is only present as TTTATTACTACG**A**T**G**GTAGCTAC in the genomic reads. Other polymorphisms that were found using genomic validation of the inferred genes are underlined. For example, GATACAG**C**GGGTACAGT was inferred by MINING-D as a variant of the macaque gene IGHD5S3*01, but the whole sequence G**G**GGATACAG**C**GGGTACAGTTAC was found in the genomic reads.

**Human**	
IGHD3-10*01 Original GTATTACTATGGTTCGGGGAGTTATTATAAC N_Var-3 GTATTACTATGGTTC**A**GGGAGTTATTATAAC	IGHD3-16*02 Original GTATTATGATTACGTTTGGGGGAGTTATCGTTATACC N_Var-0 ---TTATGATTAC**A**TTTGGGGGAGTTATCGTTAT---
**Camel**	
IGHD3*01 (Alpaca) Original GTATTACTACTGCTCAGGCTATGGGTGTTATGAC N_Var-1 ----**G**ACT**G**CT**AT**TCAGGCT**C**T**T**GGTGTTATG-- N_Var-0 ---T**G**ACTACTG**T**TCAGGCT**C**T**T**GGTGT------	IGHD2*01 (Alpaca) Original ACATACTATAGTGGTAGTTACTACTACACC N_Var-1 --ATA**T**T**G**TAGTGGT**G**GTTACT**G**CTAC--- N_Var-0 **G**CATACTATAGTGGT**G**GTTACTAC------
IGHD4*01 (Alpaca) Original TTACTATAGCGACTATGAC N_Var-1 **C**TACTATAGCGACTATG-- N_Var-0 **C**TACTATA**A**CGA**A**TATG--	IGHD6*01 (Alpaca) Original GTACGGTAGTAGCTGGTAC N_Var-2 GTACGGT**G**GTAGCTGGTAC
IGHD5*01 (Alpaca) Original AGACTACGGGTTGGGGTAC N_Var-0 ----TA**T**GGGTT-GGGTAC	
**Rhesus Macaque**	
IGHD1S39*01 Original GGTATAGTGGGAACTACAAC N_Var-0 -----AGTGGGA**G**CTAC---	IGHD3S18*01 Original GTACTGGGGTGATTATTATGAC N_Var-0 --ACTGG**A**GTGATTATTA----
IGHD5S3*01 Original GTGGATACAGTGGGTACAGTTAC N_Var-0 -**G**-GATACAG**C**GGGTACAGT---	IGHD2S11*01 Original AGAATATTGTAGTAGTACTTACTGCTCCTCC N_Var-0 --**C**--ATTGTAGT**G**GTACTTACTGCT**ATG**--
IGHD2S17*01 Original AGAATACTGTACTGGTAGTGGTTGCTATGCC N_Var-0 ----TACTGTACTGGTAGTGGTTGCTA**C**---	IGHD3S23*01 Original GTATTACTATGATAGTGGTTATTACACCCACAGCGT N_Var-0 ---TTACTATG**G**TAGTGGTTATTAC-----------
**Mouse**	
IGHD1-1*01 Original TTTATTACTACGGTAGTAGCTAC- N_Var-0 TTTATTACTACG**A**T**G**GTAGCTACg	
**Rat**	
IGHD1-3*01 Original TTTTTAACTATGGTAGCTAC N_Var-0 -TTTTAACTA**C**GGTAGCTAC	IGHD1-9*01 Original TACATACTATGGGTATAACTAC- N_Var-1 --CATACTA**C**GGGTATA**C**CTACg
IGHD1-12*02 Original TTTATTACTATGATGGTAGTTATTACTAC- N_Var-0 -TTATTACTATGATGGTA**C**TTATTACTACg	
**Rabbit**	
IGHD6-1*01 Original --------------GTTACTATAGTTATGGTTATGCTTATGCTACC N_Var-4 **G****TTACTATACTTATG**GTTA**TGC**T**G**GTTATG**C**TTATGCT**AC****C** N_Var-3 **G****TTA**------**TGCTG**GTTA**TGC**T**G**GTTATGGTTATGCT**AC****C**	IGHD1-1*01 Original GCATATACTAGTAGTAGTGGTTATTATATAC N_Var-2 GCATAT**G**CTAGTAGTAGTGGTTATTAT----

For rhesus macaques, the two novel genes inferred seem to be two variations of the same novel gene with the following sequences:

N_Gene-0                     TACAAT**T**TTTGGA**G**TGGTTAT

N_Gene-1                ATTACAAT**A**TTTGGA**C**TGGTTATTAT

The sequences of these genes found in the genomic data from different individuals of the same species are as follows:

N_Gene-0                GTATTACAAT**T**TTTGGA**G**TGGTTATTA**C**ACC

N_Gene-1                GTATTACAAT**A**TTTGGA**C**TGGTTATTA**T**ACC.

### Validation of novel D gene variations using Whole Genome Sequencing data

To validate novel genes and variations discovered by MINING-D, we downloaded genomic reads for all analyzed species and searched for the occurrences of the novel genes and variations in these reads (see details in Supplemental Note: Finding D genes in Whole Genome Sequencing Data). Since paired genomic and immunosequencing datasets were not available, genomic and immunosequencing reads came from different individuals (Table 5). We consider an inferred novel D gene or variation validated if it is present in at least 2 reads and is surrounded by RSS motifs on both sides. Table 5 provides details of the downloaded data and information about validated variations.

**Table 5:** Genomic data used for validating discovered D gene variations. The last column describes the number of datasets in which the novel sequences were found in genomic reads and the range of number of reads in which the sequences were found. For rhesus macaques, we chose only 4 datasets out of the 1318 in the NCBI project PRJNA382404 for analysis.

Species	Project	Description	Datasets	Novel variations/genes found in genomic reads	# datasets (# reads)
Human	PRJNA427604	WES of human PBMC (ESCC—cohort, China)	40	N_Var (IGHD3-10*01)-3 N_Var (IGHD3-16*02)-0	5 (8–14) 6 (30–58)
Mice	PRJEB18467	WGS of house mouse	32	N_Var (IGHD1-1*01)-0	19 (1–10)
Bactrian Camel	PRJNA276064	WGS of Old world camels	7	N_Var(IGHD2*01)-0 N_Var(IGHD2*01)-1 N_Var(IGHD3*01)-0 N_Var(IGHD3*01)-1 N_Var(IGHD4*01)-0 N_Var(IGHD4*01)-1 N_Var(IGHD5*01)-0 N_Var(IGHD6*01)-2	2 (2) 6 (4–17) 2 (2–6) 7 (1–16) 2 (4–5) 6 (7–15) 6 (2–13) 7 (5–21)
Rhesus Macaque	PRJNA382404	WGS of rhesus macaques	4/1318	N_Gene-1 N_Gene-1-0 N_Var (IGHD1S39*01) N_Var (IGHD3S18*01) N_Var (IGHD5S3*01) N_Var (IGHD2S11*01) N_Var (IGHD2S17*01) N_Var (IGHD3S23*01)	4 (9–27) 2 (8–9) 1 (18) 2 (8–21) 4 (13–28) 1 (6) 4 (8–30) 3 (12–24)
Wistar Rats	PRJNA479378	WGS of wistar rats	10	N_Var (IGHD1-12*02)-0 N_Var (IGHD1-3*01)-0 N_Var (IGHD1-9*01)-1	10 (1–9) 10 (2–18) 10 (2–8)
Rabbit	PRJNA242290	WGS of rabbits and hares	24	N_Var (IGHD1-1*01)-2 N_Var (IGHD6-1*01)-3 N_Var (IGHD6-1*01)-4	23 (1–14) 19 (1–9) 11 (1–6)

### Usage of D genes

**Fig 4:**
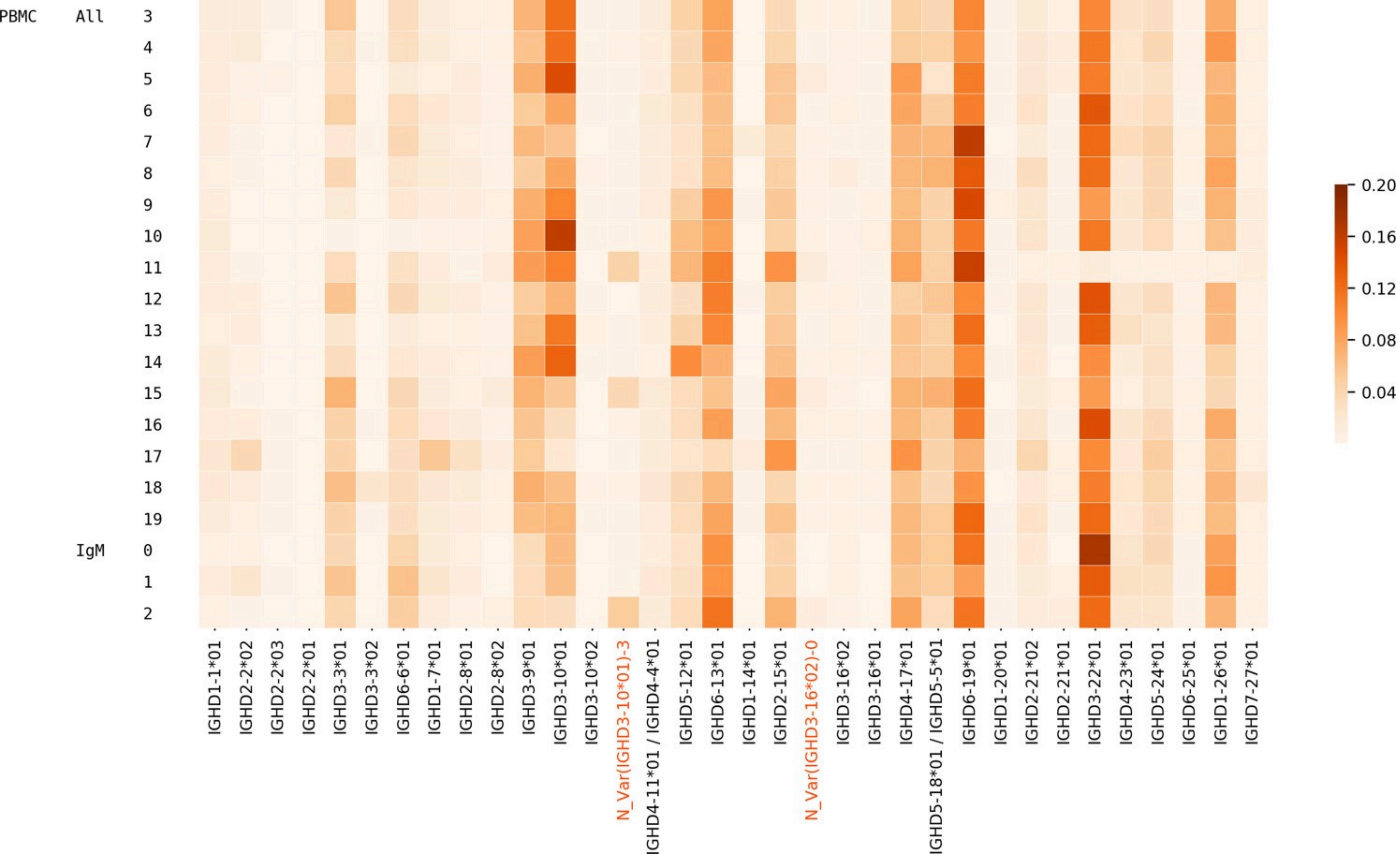
Usage of various known and novel genes in various Healthy datasets. Each row corresponds to a different dataset described by the three leftmost columns. The first column denotes the tissue, i.e., PBMC, the second column denotes the isotype (IgM or all/unsorted), and the numbers in the third column represent different individuals. The color in each cell represents the proportion of traceable CDR3s that were formed by a gene on the x-axis in the dataset corresponding to the y-axis. Validated novel variations are highlighted on the x-axis.

We analyzed the usage of all IMGT D genes and validated novel genes/variations in Healthy PBMC datasets. 54.1% of CDR3s on average were traceable. The usage of all genes is mostly consistent across individuals, although there are a few deviations for certain individuals owing to their germline variations (Fig 4). Potential deletion polymorphisms involving multiple contiguous IGHD genes, as reported in the past [13, 43], can also be seen in Fig 4. Donor 10 likely has a deletion of genes D3-3 – D6-6 and Donor 11 likely has a deletion covering genes D3-22, D5-24, and D1-26.

**Fig 5:**
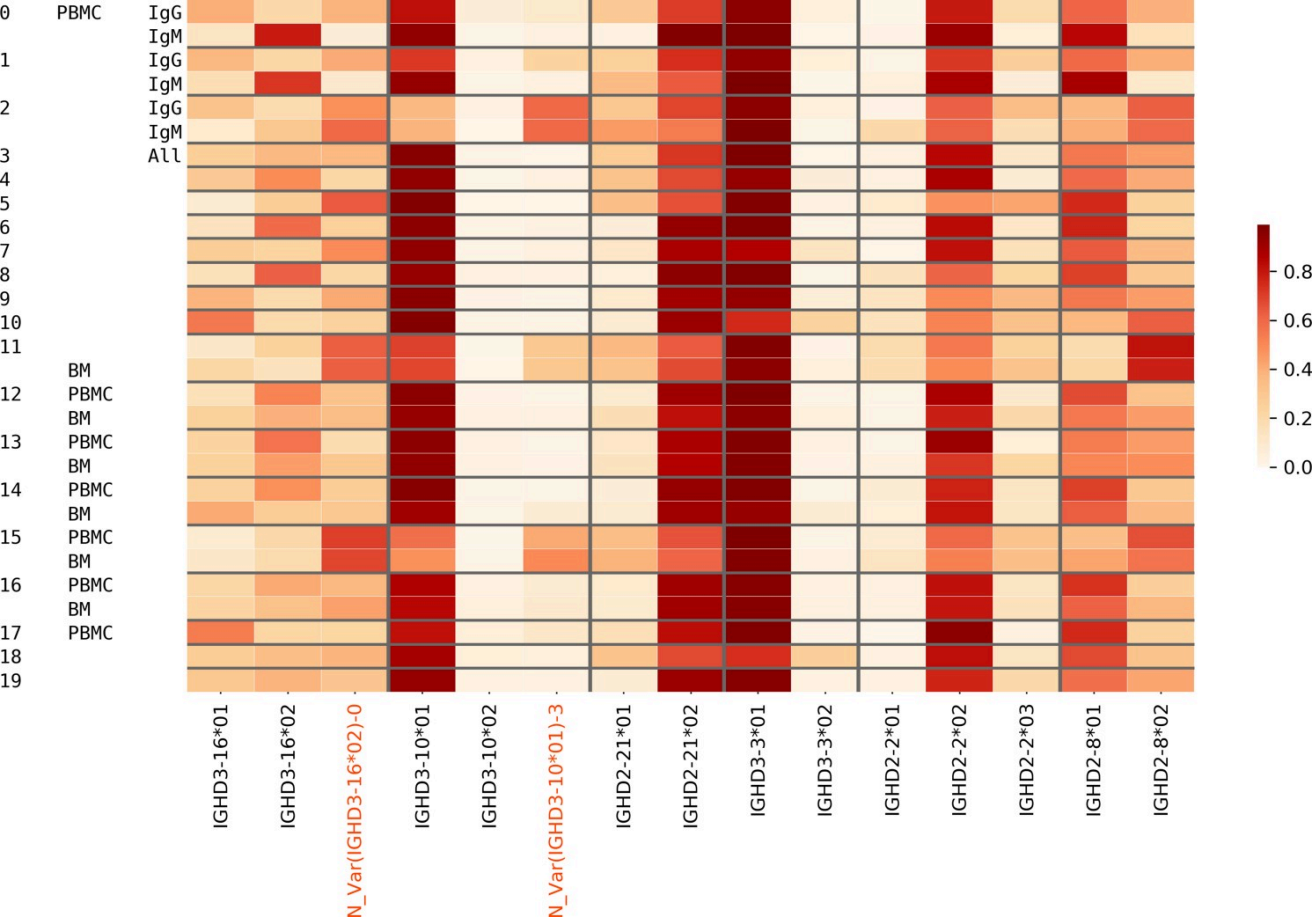
Usage of variants of D genes in Healthy PBMC BM datasets. Gray lines separate the plot such that each subplot corresponds to one gene and its variants. Each cell in a subplot represents the proportion of the usage of a variant with respect to the total usage of all variants. Thus, in every subplot, the sum of all rows is 1. The columns on the y-axis tick labels represent the individual, the tissue, and the isotype, respectively.

To analyze the relative usage of a variant of a D gene (known or novel) against other variants of the same gene, we also included the bone marrow datasets and plotted the variant usage in Healthy PBMC BM (Table 1) datasets (Fig 5). We found that extensive SHMs in IgG repertoires may lead to misclassification of alleles for some genes e.g. IGHD3-16 and IGHD2-8. For these genes, we accurately compute the D gene allele usage using decoy alleles as explained in the next subsection.

**Fig 6:**
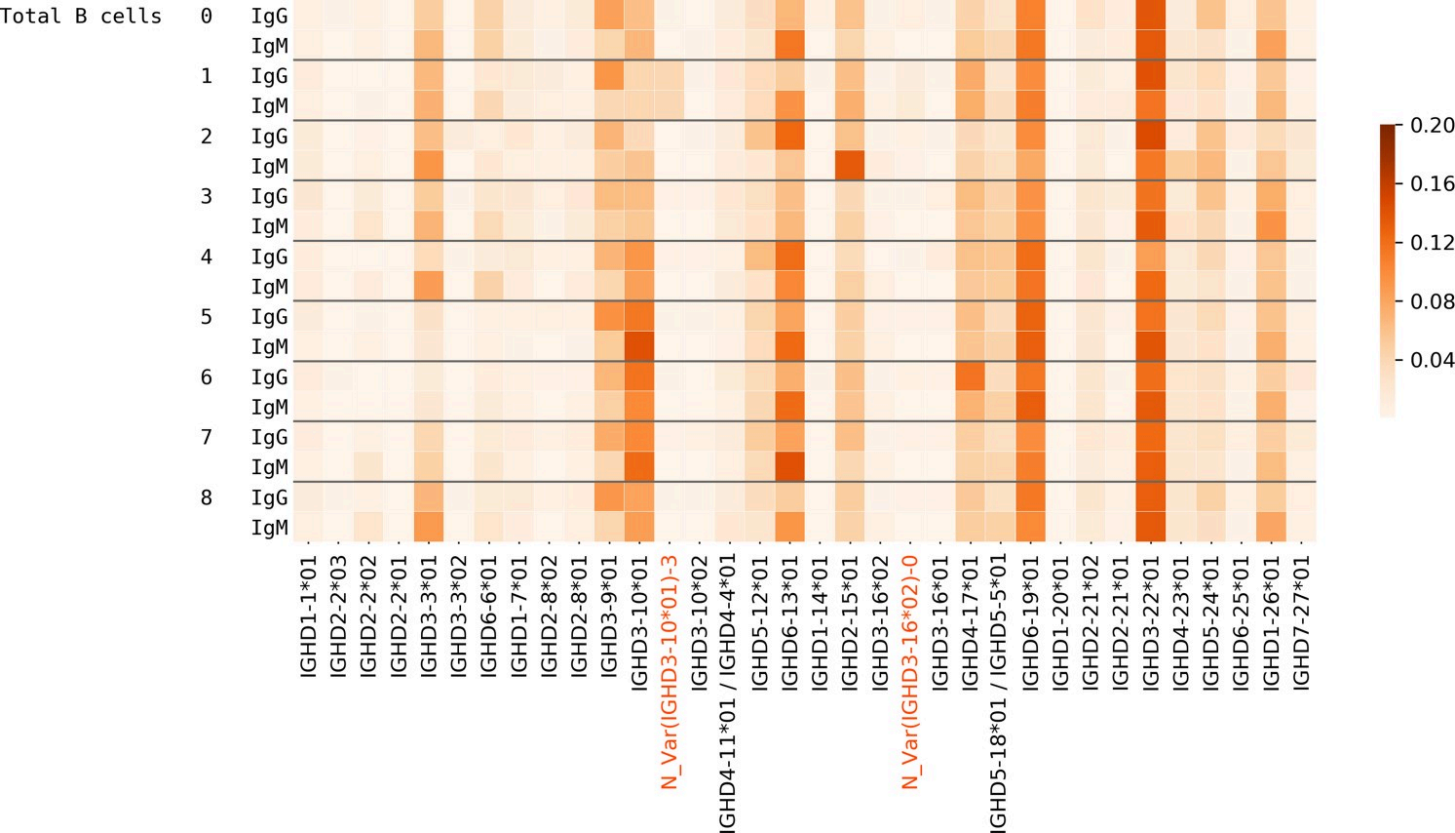
Usage of D genes in the Hepatitis B datasets corresponding to the IgG and IgM isotypes from various individuals.

In the Stimulated datasets, some differences were seen in the usage of D genes in IgG and IgM datasets (see Supplemental Note: D gene usage). For instance, in the Hepatitis B datasets, 65.4% and 45.9% CDR3s were traceable on average in datasets corresponding to IgM and IgG isotypes, respectively. The usage of some genes differs in datasets corresponding to IgG and IgM isotypes from the same individual (Fig 6). For example, genes IGHD1-26*01, IGHD6-13*01, and IGHD3-3*01 appear to be used more in the IgM datasets for most individuals whereas IGHD3-9*01 is used more in the IgG datasets.

### Accurate computation of the D gene allele usage

High SHM rate in IgG datasets can lead to inaccurate labeling of CDR3s in terms of the D gene allele used. Fig 5 shows a checkered pattern (particularly in the usage of genes IGHD2-8 and IGHD3-16 in IgM and IgG datasets) for individuals 0 and 1—while IgM datasets show homozygous state of IGHD3-16 formed by allele 2, IgG datasets show heterozygous state of IGHD3-16 formed by the known allele 1 and the novel allele N_Var-0. This is because the estimated usage of a D gene depends not only on the sequence of that gene but also on the sequences of other genes. If two genes have very similar sequences and only one of them is present in the database, the CDR3 sequences originating from both the genes will get assigned to the one that is present in the database.

When there are many SHMs, some hypermutated reads (CDR3s) can get assigned to one of the allelic variations if only a few (2–3, usually germline) are present in the database. Since the usage of alleles of a D gene is calculated in terms of proportion of the total usage of the D gene, even a small number of hypermutated CDR3s that got assigned to a wrong allele (because not all possible variations of the gene were in the database) can show up as a considerable proportion of the total usage, particularly if the total usage is small. This is what happened in the cases of genes IGHD3-16 and IGHD2-8 (Fig 5).

**Fig 7:**
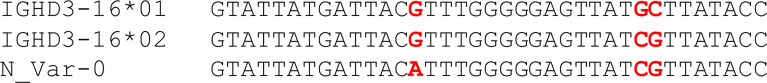
Alleles of the gene IGHD3-16.

To circumvent this issue, we added artificial alleles of IGHD2-8, IGHD3-16, and IGHD3-10 to the D genes database to check if all the CDR3s that were assigned to alleles in the IgG datasets for subjects 0, 1, and 2 (Fig 5) would still be assigned to the same alleles in the presence of these false variations. We added 61 alleles for IGHD3-16 that are possible with mutations at the highlighted sites in Fig 7.

**Fig 8:**
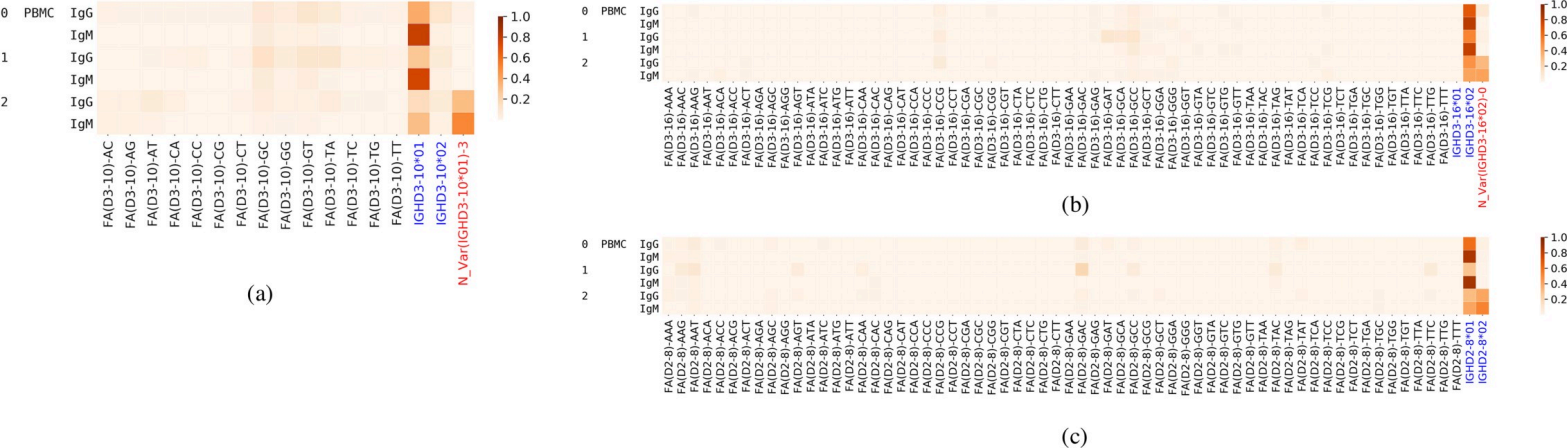
**Allelic variant usage for genes IGHD3-10 (a), IGHD3-16 (b) and IGHD2-8 (c).** FA stands for false alleles. The alleles listed in IMGT are shown in blue. Novel inferred genes are shown in red.

The results of D gene labeling are shown in Fig 8. Most of the CDR3 reads that were falsely assigned were distributed among the false alleles whereas the ones which were correctly assigned did not.

For genes IGHD3-16 and IGHD2-8, the total usage was much smaller than other genes e.g. IGHD3-10. That is why the CDR3s incorrectly assigned to alleles made up a considerable proportion of the total number of CDR3s that were assigned to all alleles of the gene. Fig 8(A) illustrates that results for genes with a slightly higher usage are similar to results in Fig 5.

### Haplotyping heterozygous V genes using D genes

**Fig 9:**
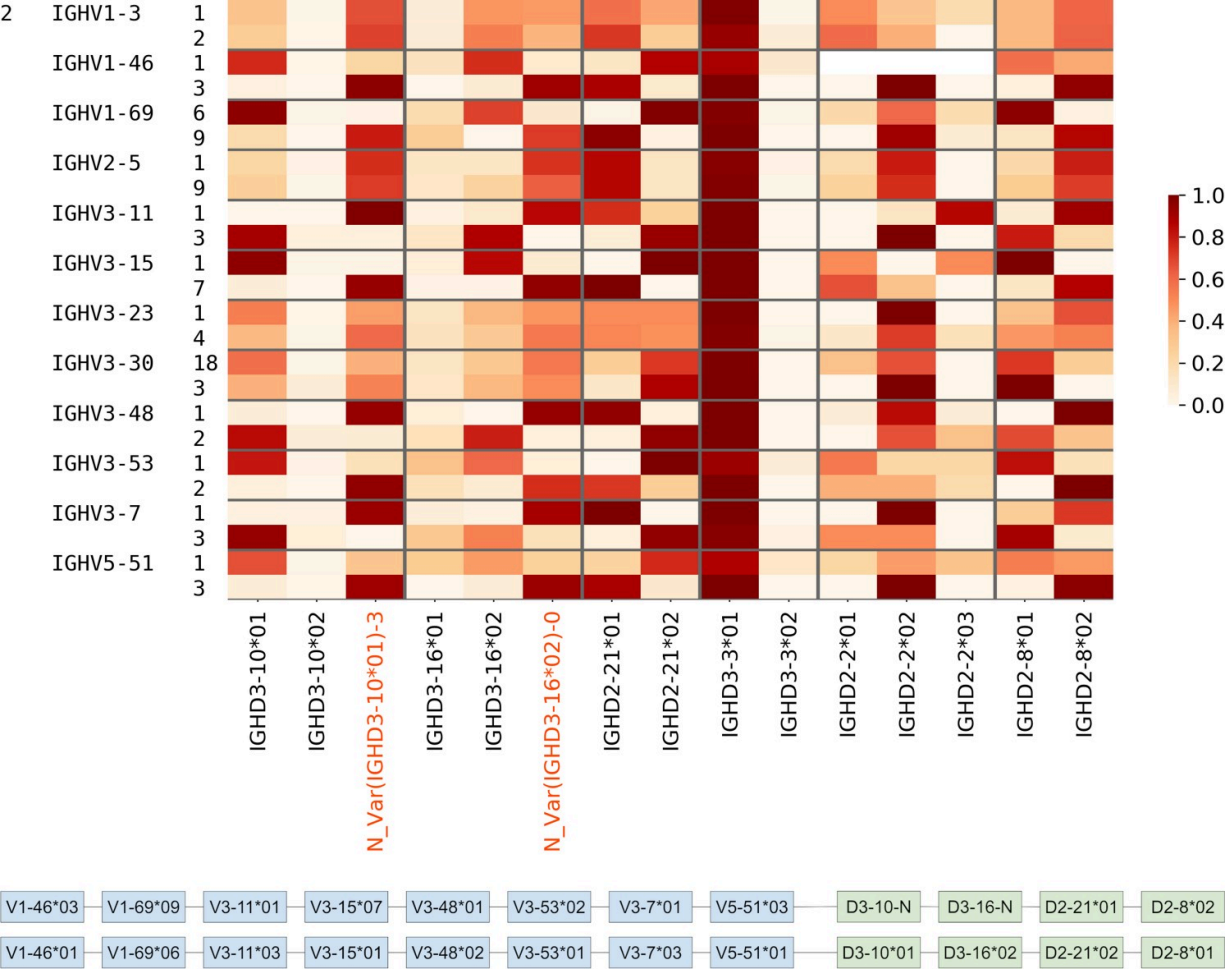
Haplotypes of IGHV genes for individual 2 from Healthy PBMC datasets (Fig 4). (Upper) Joint usage of V and D gene alleles. Alleles of V genes are shown at the third column on the left. A cell corresponding to allele X of gene V and allele Y of gene D shows the number of distinct CDR3s derived from alleles X, Y normalized by the total number of distinct CDR3s derived from allele X and gene D. Gray lines separate the plot such that each subplot corresponds to one gene and its alleles. (Lower) Haplotypes of IGHV are inferred according to the pairings of alleles of V (blue) and D (green) genes supported by the maximum number of CDR3s.

**Fig 10:**
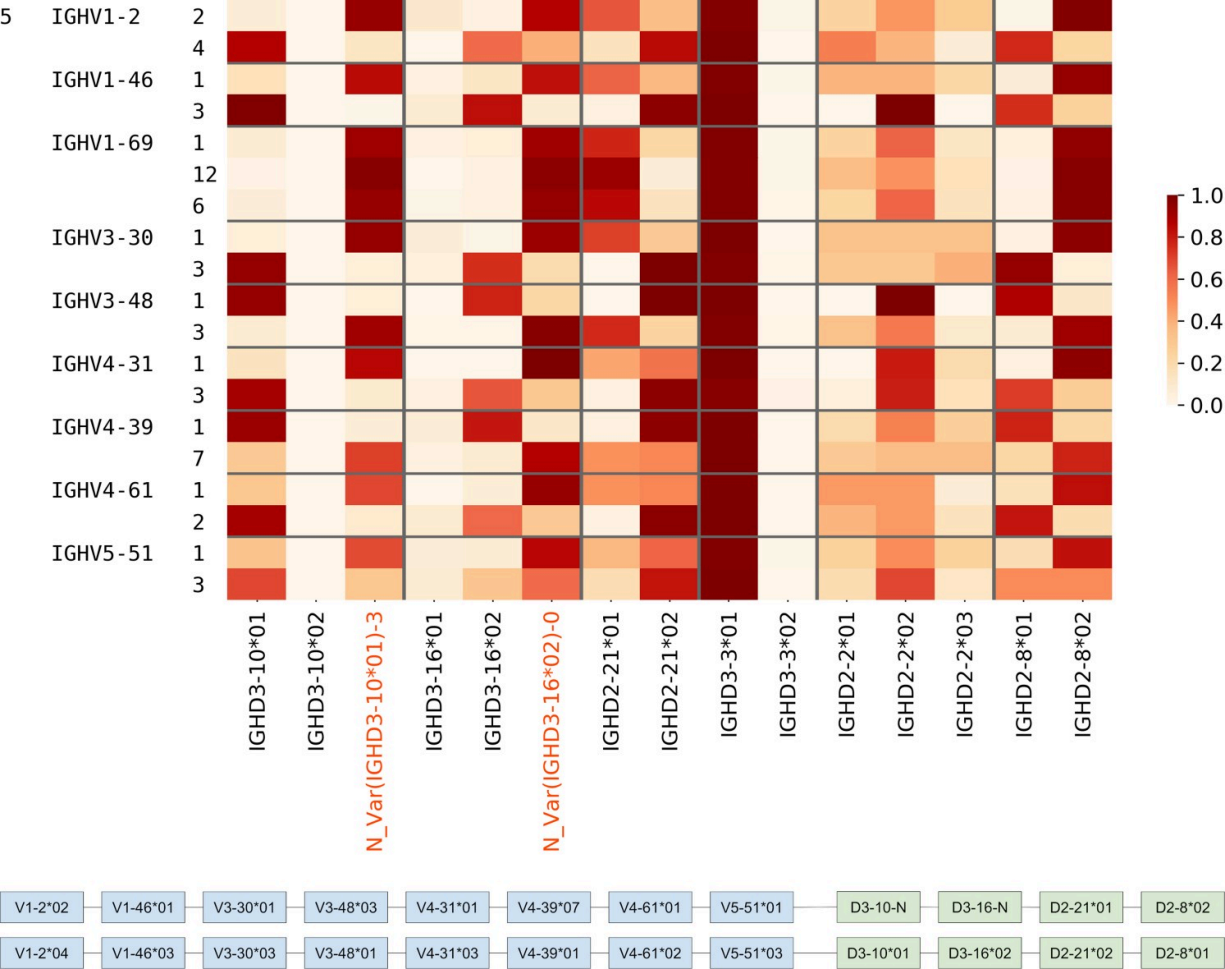
Haplotypes of IGHV genes for individual 5 from the Intestinal datasets (Figure J) (see legend for Fig 9).

To support the inferences of the novel alleles found in the Healthy datasets, we used them for inference of haplotypes of V genes. Haplotype inference, whenever subjects are heterozygous with respect to some genes, can lend support to the identification of novel alleles of the germline genes [44]. We analyzed two Rep-seq datasets corresponding to individual 2 from Healthy PBMC datasets (Fig 4) and individual 5 from the Intestinal datasets (see Supplemental Note: D gene usage, Figure J). For each individual, we selected V genes that are present in corresponding Rep-seq datasets in the form of at least two allelic variants. To minimize the impact of the sample preparation artifacts and SHMs, we ignored alleles with low usage (<1000 distinct CDR3s). As a result, we selected 12 and 9 heterozygous V genes for individuals 2 and 5, respectively (Table 6). Afterward, we extracted distinct CDR3s corresponding to each of the selected alleles and identified D genes in them. The joint usage of V and D gene alleles allows us to identify haplotypes of V genes and 4 heterozygous D genes (including novel alleles of IGHD3-10 and IGHD3-16) in individuals 2 (Fig 9) and 5 (Fig 10).

**Table 6:** Abundant heterozygous IGHV genes in individual 2 from Healthy PBMC datasets (Fig 4) and individual 5 from the Intestinal datasets (Figure J). For individual 2, only the IgM datasets were used. For individual 5, only the naive datasets were used.

V gene	Allele	# distinct CDR3s	
		Individual 2, Fig 4	Individual 5, Figure J
IGHV1-2	2	-	4152
	4	-	1299
IGHV1-3	1	7166	-
	2	2175	-
IGHV1-46	1	2154	2717
	3	1058	1445
IGHV1-69	1	-	7872
	6	8820	5194
	9	1914	-
	12	-	3052
IGHV2-5	1	2667	-
	9	2621	-
IGHV3-7	1	2841	-
	3	2276	-
IGHV3-11	1	2569	-
	3	1114	-
IGHV3-15	1	2263	-
	7	2339	-
IGHV3-23	1	1428	-
	4	26138	-
IGHV3-30	1	-	2621
	3	1617	2784
	18	6245	-
IGHV3-48	1	3559	1581
	2	3538	-
	3	-	2753
IGHV3-53	1	2495	-
	2	1048	-
IGHV4-31	1	-	1488
	3	-	8183
IGHV4-39	1	-	6732
	7	-	3448
IGHV4-61	1	-	2828
	2	-	7271
IGHV5-51	1	9561	17635
	3	2143	4082

We could not infer haplotypes using IGHD2-2 gene because differences between its alleles are concentrated in the start of the gene that is often truncated. We also did not use gene IGHD3-3 that is homozygous in both individuals. In individual 2, we could not infer haplotypes for the 4 out of 12 selected V genes: IGHV1-3, IGHV2-5, IGH3-23, and IGHV3-30. In individual 5, we could not infer haplotypes of IGHV1-69. We assume that it may be caused by the presence of these genes in several copies and SHMs (in individual 2).

Within an individual, haplotypes of the remaining V genes are consistent across all heterozygous D genes. Thus, haplotyping results lend additional support for novel alleles of D genes and prove that heterozygous D genes can be used for haplotyping the IGH locus.

### Overused D genes in datasets specific to a health condition, tissue, and/or cell type

To see any potential association between the usage of a D gene and an environment (a health condition, a tissue, or a cell type), we analyzed the usage of D genes in Stimulated and Tissue-specific datasets. We use the gene usage profiles in the Healthy PBMC datasets as a reference and compare the D gene usage profiles in other datasets.

**Fig 11:**
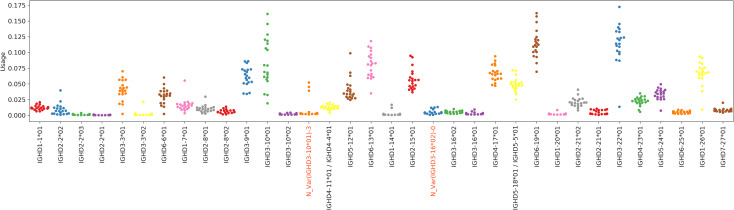
D gene usage in all Healthy datasets. Each point above a gene represents a Healthy Human PBMC dataset. To distinguish usages of different genes, adjacent genes are represented by different colors.

**Fig 12:**
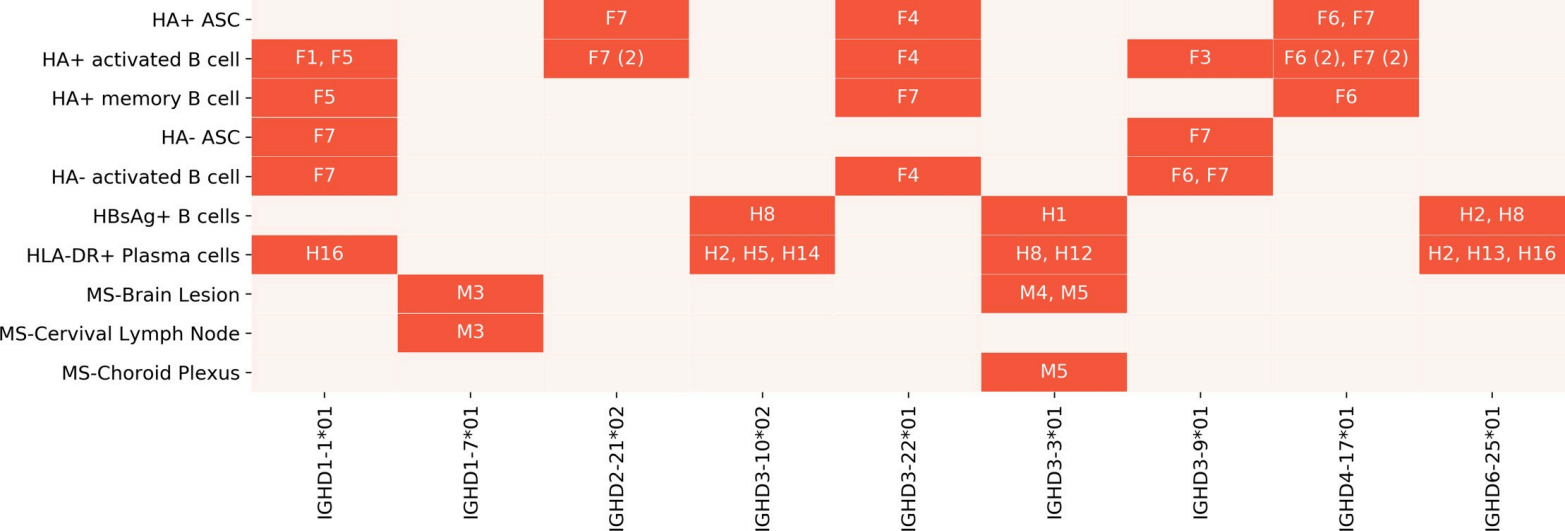
Summary of overused genes in Stimulated datasets. The datasets in which each gene is overused are highlighted and annotated with the corresponding individuals. Subjects were prefixed with a letter corresponding to the project–“F” for Flu Vaccination, “M” for Multiple Sclerosis, and “H” for Hepatitis B Vaccination. Some genes were overused in multiple datasets from the same and/or different individuals. The number in parentheses shows the number of datasets from the same individual in which the gene was overused.

**Fig 13:**
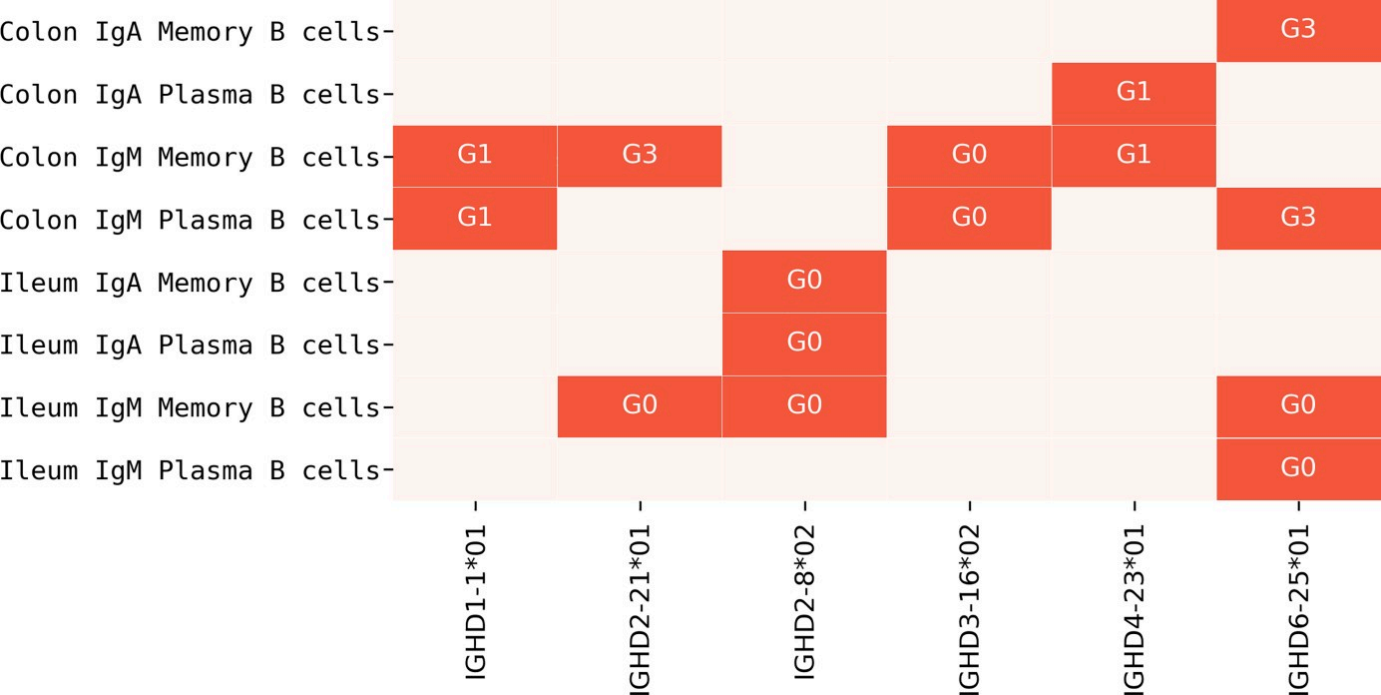
Summary of overused genes in Intestinal datasets. The datasets in which each gene is overused are highlighted and annotated with the corresponding individuals. The subjects in the Intestinal Repertoire project were prefixed with “G”.

We say that a gene is *overused* in a dataset if the usage of the gene in that dataset is at least twice the maximum usage of that gene in all Healthy PBMC datasets. The ratio of usage of an overused gene to the maximum usage in Healthy PBMC datasets is referred to as *over-usage*. The usages of all IMGT D genes and validated novel variations in Healthy Human PBMC datasets are shown in Fig 11. Details on the genes overused in the Flu Vaccination datasets are shown in Table 7, and overused genes in other Stimulated and Tissue-specific datasets are shown in Supplemental Note: Over-usage of D Genes. In total, 9 genes were overused in at least 2 datasets of the same type in all Stimulated datasets (Fig 12), and 6 genes were overused in at least 2 datasets from the Intestinal datasets (Fig 13). These results suggest potential associations between the usage of a D gene and a health condition, tissue, or cell type, although it is difficult to infer statistically significant associations with such a small sample size.

**Table 7:** Overused genes in Flu Vaccination datasets. Since the number of datasets in PRJNA324093 is much greater than in other projects, only genes that are overused in at least three different datasets are shown. The over-usage of a gene in a dataset is also shown. For example, the usage of IGHD1-1*01 in HA+ activated B cells for donor 1 is 3.7 times the maximum usage in all Healthy Human datasets.

Gene	Cell type	Donor	Over-usage
IGHD1-1*01	HA+ activated B cell	1	3.7x
		5	13.5x
	HA+ memory cells		5.8x
	HA- activated B cell	7	2.4x
	HA- ASC		4.3x
IGHD2-21*02	HA+ activated B cell	7	8.8x
			6.4x
	HA+ ASC		4.4x
IGHD3-22*01	HA+ activated B cell	4	2.2x
	HA+ ASC		3.4x
	HA- activated B cell		4.4x
	HA+ memory B cell	7	3.2x
IGHD3-9*01	HA+ activated B cell	3	2.0x
	HA- activated B cell	6	2.2x
		7	2.0x
	HA- ASC		2.1x
IGHD4-17*01	HA+ activated B cell	6	9.5x
			8.6x
		7	4.0x
			4.0x
	HA+ ASC	6	9.7x
		7	6.7x
	HA+ memory B cell	6	7.2x

### Usage of D genes in the Mouse datasets

**Fig 14:**
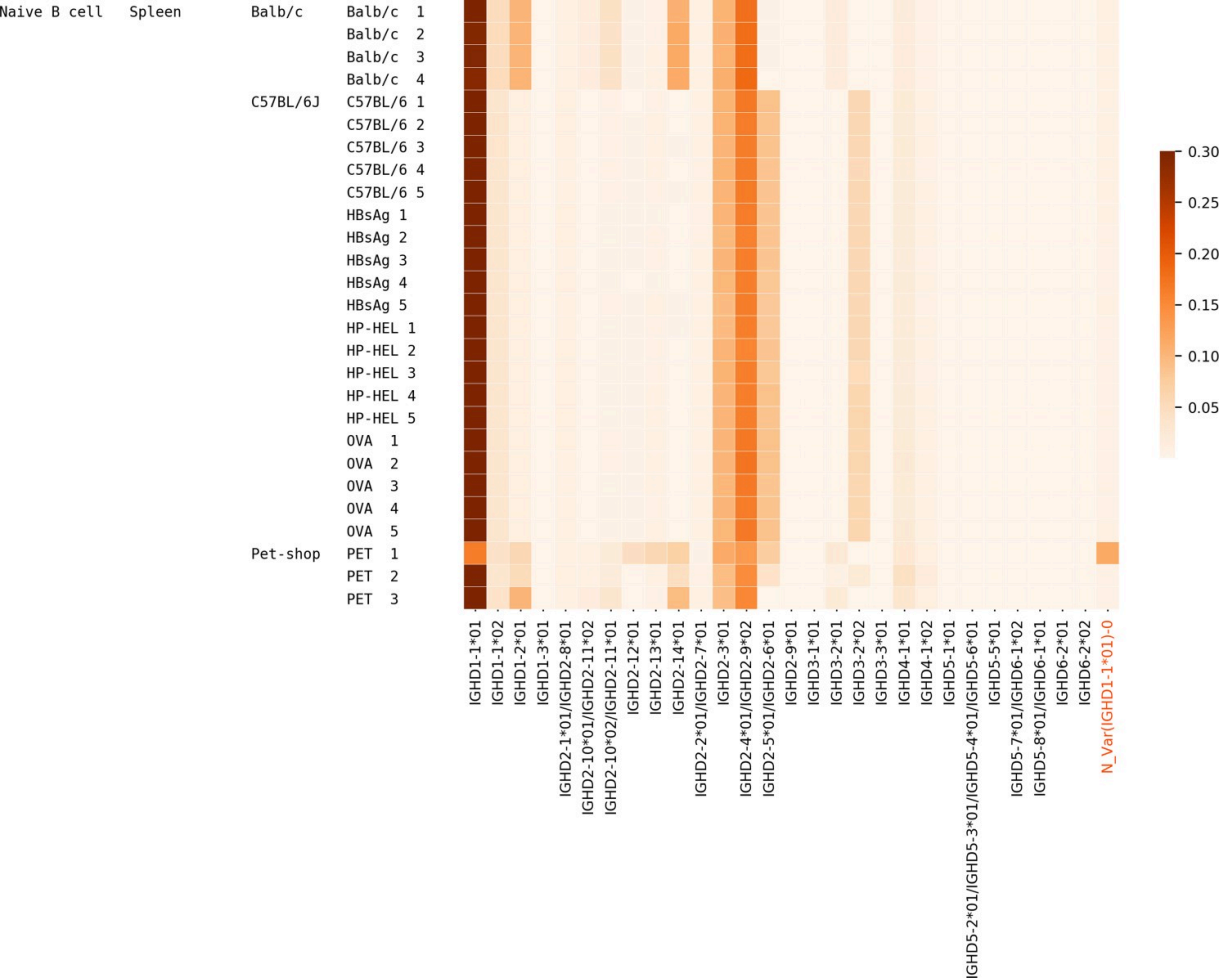
Usage of various known and novel genes/variations in MICE datasets. Columns on the left represent cell type, tissue, strain, and individual respectively. OVA, HP-HEL, and HBsAg in the right most column represent the C57BL/6J mice immunized with OVA, HP-HEL, and HBsAg, respectively. For example, OVA 3 represents the C57BL/6J mouse number 3 that was immunized with OVA.

57.4% of CDR3s on average were traceable in each dataset. Fig 14 shows the usage of mouse D genes (annotated in IMGT mice and one validated novel variant) in the datasets corresponding to naive B cells of various mice (see also Supplementary Note: D gene usage). The usage of genes among individuals of the same strain is similar. In contrast, the usage of genes among individuals of different strains (Balb/c, C57BL/6J, pet mice) is very different. The gene usages in two of the three pet shop mice (Pet 1 and Pet 2) of unknown strains show a departure from both Balb/c and C57BL/6J strains.

**Fig 15:**
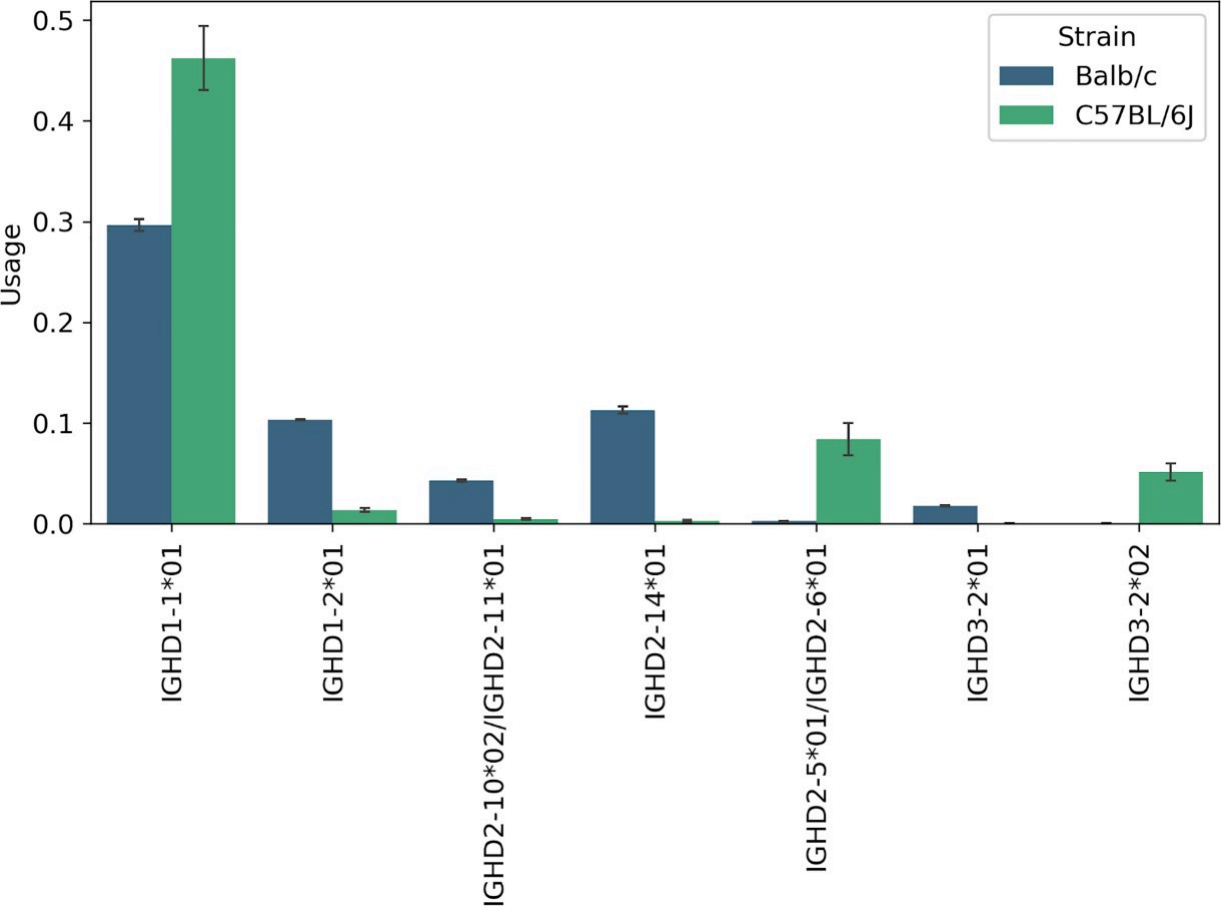
Genes with differential usage in Balb/c and C57BL/6J strains. Except IGHD1-1*01, all genes were inferred only in one strain.

The genes with differential usage in strains Balb/c and C57BL/6J are shown in Fig 15. Although the gene IGHD1-1*01 is only listed as a Balb/c gene in the IMGT database, we inferred it in both strains. We inferred genes IGHD1-2*01, IGHD2-10*01/IGHD2-11*01, IGHD2-14*01, and IGHD3-2*01 from only the Balb/c datasets–among these, three of them are listed as Balb/c genes in IMGT whereas IGHD2-14*01 is listed only as a 129/Sv gene. Genes IGHD3-2*02 and IGHD2-5*01/IGHD2-6*01 were inferred from only the C57BL/6J datasets. IGHD3-2*02 is listed as a C57BL/6J gene in the IMGT database. The genes IGHD2-5*01 and IGHD2-6*01 have the same sequence and are listed under the CB.20 strain and C57BL/6J strain, respectively, in the IMGT database. The results suggest that other than the novel variation that is not listed in the IMGT database for any strain, there are some genes which are listed in the IMGT database of some strains but were also inferred from other strains.

### Usage of D genes in the camel, macaque, and rat datasets

31.7%, 52.6%, and 54.3% of CDR3s were traceable on average in the Camel, Macaque, and Rat datasets, respectively (see Supplemental Note: D Gene Usage). For camels, the D gene usage profiles were slightly different for the VH and the VHH isotypes within individuals (Figure O in S1 Notes). For rats, genes belonging to the IGHD2 and IGHD3 families were used much less than in other gene families (Figure P in S1 Notes). D genes with the highest usage among datasets of a species are shown in Supplemental Note: Highly Used D Genes in Non-human Datasets.

## Discussion

Although inference of *personalized* immunoglobulin V, D, and J genes is now recognized as an important step in the analysis of immunosequencing data [26], inference of D genes presents additional difficulties as compared to inference of V and J genes [5]. Indeed, since D genes undergo exonuclease removals during VDJ recombination (and since they are much shorter than V and J genes), the alignment-based techniques used for V and J gene reconstruction do not work for D gene reconstruction.

Since the most abundant *k*-mers of CDR3s usually originate from D genes, iterative recruitment and extension of abundant *k*-mers in CDR3s (implemented in IgScout [28]) results in *de novo* reconstruction of many germline D genes. The performance of IgScout depends on the value of *k*: selecting a large *k* results in missing short D genes, but selecting a small *k* presents a danger of recruiting *k*-mers that belong to multiple D genes and thus missing some of these genes or producing inaccurate results. For inference of human D genes, IgScout uses *k* = 15 since all 15-mers in known human D genes are unique and all human D genes but one are at least 15 nucleotides long. However, it is unclear how to select the parameter *k* for species with still unknown sets of D genes.

The described MINING-D algorithm does not assume previous knowledge of the lengths of D genes and, unlike IgScout, considers multiple extensions of *k*-mers and thus can use short *k*-mers as seeds (the default value *k* = 10 does not exceed the length of all known D genes). Benchmarking MINING-D on simulated datasets demonstrate high accuracy of the inferred D genes (Supplemental Note: Benchmarking MINING-D on simulated CDR3s).

We applied MINING-D to 588 Rep-seq datasets from various species and inferred 38, 24, 16, 25, 13, and 18 D genes using human, mouse, rat, macaque, camel, and rabbit datasets, respectively. 25 (13), 18 (6), 12 (4), 17 (8), 1 (12), and 3 (15) of human, mouse, rat, macaque, camel, and rabbit D genes were known (novel), respectively. We additionally validated the novel genes and variations using genomic data. Unfortunately, since paired Rep-seq and WGS datasets are currently not available, we could not validate the inferred D genes with genomic data taken from the same individuals. Instead, we downloaded 117 publicly available WGS datasets from different individuals and searched for occurrences of the inferred novel D genes and variations. In total, we validated 25 of the 58 novel D genes/variations. There are multiple reasons why some of the inferred D genes were not validated, e.g., it is difficult to validate a rare allele of a D gene (since paired WGS and Rep-Seq data are not available), inferred gene may be a result of highly abundant SHM rather than a real D gene, etc. We also validated novel alleles of human D genes using haplotyping of heterozygous V genes and showed that haplotypes computed using novel and known D genes are consistent. Additionally, we benchmarked MINING-D on TCR datasets (Supplemental Note: Benchmarking MINING-D on TCR datasets).

Finally, we analyzed the usage of inferred D genes in diverse Rep-seq datasets and found that it is highly conservative in healthy humans. To see whether a gene is overused in some specific datasets corresponding to a health condition, tissue, and/or cell type, we compared the usage in these datasets against the usage in Healthy Human PBMC datasets as a reference. Based on the results of this comparison, we propose potential associations between some D genes and a health condition, tissue, and/or cell type, albeit the small sample size keeps us from inferring statistically significant associations. In total, we found 9 overused genes among the Flu Vaccination, Multiple Sclerosis, or Hepatitis B Vaccination datasets.

We also analyzed the D gene usage in two mouse strains (Balb/c and C57BL/6J) and demonstrated that the usage of genes among individuals of the same (different) strains was very similar (different). For example, the gene IGHD1-1*01 (which was inferred in both strains) had a much higher usage in the C57BL/6J strain. Since this gene is only listed as a Balb/c gene in the IMGT database, we propose to add it to the database of C57BL/6J genes as well. Similarly, we propose to add IGHD2-14*01 to the Balb/c genes, which is only listed as a 129/Sv gene in the IMGT database.

We demonstrated that high SHM rate may result in erroneous inferences that represent abundant hypermutations rather than novel alleles of D genes (see Supplemental Note: Benchmarking MINING-D on simulated CDR3s). Therefore, inference of novel alleles of D genes (as well as other immunoglobulin genes) must be done from data minimally affected by SHMs (such as naive or IgM / IgD Rep-Seq data) with a follow-up validation of the inferred alleles by genomic data. Using MINING-D, we inferred and validated 25 novel genes/variations in humans, mice, camels, rhesus macaques, rats, and rabbits. We argue that validated novel variations of D genes must be added to standard databases of germline genes to make the analysis of the antibody repertoire data more accurate. In addition, we also analyzed the usage of the known and validated novel D genes in the VDJ recombination process and found that although the gene usage is similar in PBMCs from healthy individuals, we see some deviations in datasets that are antigen specific. Although, associations between the usage of a D gene and an antigen could not be established due to the low number of samples with a specific data type, our study suggests directions for future research.

## Supporting information

S1 Notes (PDF)

## References

[1] Cooper MD. The early history of B cells. Nat Rev Immunol. 2015;15(3):191–7. Epub 2015/02/06. 10.1038/nri3801 . 25656707

[2] Tonegawa S. Somatic generation of antibody diversity. Nature. 1983;302(5909):575–81. 10.1038/302575a0 . 6300689

[3] Turchaninova MA, Davydov A, Britanova OV, Shugay M, Bikos V, Egorov ES, et al High-quality full-length immunoglobulin profiling with unique molecular barcoding. Nat Protoc. 2016;11(9):1599–616. Epub 2016/08/04. 10.1038/nprot.2016.093 . 27490633

[4] Wang Y, Jackson KJ, Sewell WA, Collins AM. Many human immunoglobulin heavy-chain IGHV gene polymorphisms have been reported in error. Immunol Cell Biol. 2008;86(2):111–5. Epub 2007/11/27. 10.1038/sj.icb.7100144 . 18040280

[5] Ralph DK, Matsen FA. Per-sample immunoglobulin germline inference from B cell receptor deep sequencing data. 2017. arXiv:1711.05843v2. 10.1371/journal.pcbi.1007133 PMC6675132 31329576

[6] Yaari G, Uduman M, Kleinstein SH. Quantifying selection in high-throughput Immunoglobulin sequencing data sets. Nucleic Acids Res. 2012;40(17):e134 Epub 2012/05/27. 10.1093/nar/gks457 22641856 PMC3458526

[7] McCoy CO, Bedford T, Minin VN, Bradley P, Robins H, Matsen FA. Quantifying evolutionary constraints on B-cell affinity maturation. Philos Trans R Soc Lond B Biol Sci. 2015;370(1676). 10.1098/rstb.2014.0244 26194758 PMC4528421

[8] Cui A, Di Niro R, Vander Heiden JA, Briggs AW, Adams K, Gilbert T, et al A Model of Somatic Hypermutation Targeting in Mice Based on High-Throughput Ig Sequencing Data. J Immunol. 2016;197(9):3566–74. Epub 2016/10/05. 10.4049/jimmunol.1502263 27707999 PMC5161250

[9] Watson CT, Breden F. The immunoglobulin heavy chain locus: genetic variation, missing data, and implications for human disease. Genes Immun. 2012;13(5):363–73. Epub 2012/05/03. 10.1038/gene.2012.12 . 22551722

[10] Parameswaran P, Liu Y, Roskin KM, Jackson KK, Dixit VP, Lee JY, et al Convergent antibody signatures in human dengue. Cell Host Microbe. 2013;13(6):691–700. 10.1016/j.chom.2013.05.008 23768493 PMC4136508

[11] Chang CJ, Chen CH, Chen BM, Su YC, Chen YT, Hershfield MS, et al A genome-wide association study identifies a novel susceptibility locus for the immunogenicity of polyethylene glycol. Nat Commun. 2017;8(1):522 Epub 2017/09/12. 10.1038/s41467-017-00622-4 28900105 PMC5595925

[12] Boyd SD, Liu Y, Wang C, Martin V, Dunn-Walters DK. Human lymphocyte repertoires in ageing. Curr Opin Immunol. 2013;25(4):511–5. Epub 2013/08/28. 10.1016/j.coi.2013.07.007 23992996 PMC4811628

[13] Kidd MJ, Chen Z, Wang Y, Jackson KJ, Zhang L, Boyd SD, et al The inference of phased haplotypes for the immunoglobulin H chain V region gene loci by analysis of VDJ gene rearrangements. J Immunol. 2012;188(3):1333–40. Epub 2011/12/28. 10.4049/jimmunol.1102097 22205028 PMC4734744

[14] Avnir Y, Watson CT, Glanville J, Peterson EC, Tallarico AS, Bennett AS, et al IGHV1-69 polymorphism modulates anti-influenza antibody repertoires, correlates with IGHV utilization shifts and varies by ethnicity. Sci Rep. 2016;6:20842 Epub 2016/02/16. 10.1038/srep20842 26880249 PMC4754645

[15] Lefranc MP, Giudicelli V, Ginestoux C, Jabado-Michaloud J, Folch G, Bellahcene F, et al IMGT, the international ImMunoGeneTics information system. Nucleic Acids Res. 2009;37(Database issue):D1006–12. Epub 2008/10/31. 10.1093/nar/gkn838 18978023 PMC2686541

[16] Collins AM, Wang Y, Roskin KM, Marquis CP, Jackson KJ. The mouse antibody heavy chain repertoire is germline-focused and highly variable between inbred strains. Philos Trans R Soc Lond B Biol Sci. 2015;370(1676). 10.1098/rstb.2014.0236 26194750 PMC4528413

[17] Muyldermans S, Smider VV. Distinct antibody species: structural differences creating therapeutic opportunities. Curr Opin Immunol. 2016;40:7–13. Epub 2016/02/27. 10.1016/j.coi.2016.02.003 26922135 PMC4884505

[18] de los Rios M, Criscitiello MF, Smider VV. Structural and genetic diversity in antibody repertoires from diverse species. Curr Opin Struct Biol. 2015;33:27–41. Epub 2015/07/17. 10.1016/j.sbi.2015.06.002 . 26188469 PMC7039331

[19] Luo S, Yu JA, Li H, Song YS. Worldwide genetic variation of the IGHV and TRBV immune receptor gene families in humans. Life Sci Alliance. 2019;2(2). Epub 2019/02/26. 10.26508/lsa.201800221 30808649 PMC6391684

[20] Yu Y, Ceredig R, Seoighe C. A Database of Human Immune Receptor Alleles Recovered from Population Sequencing Data. J Immunol. 2017;198(5):2202–10. Epub 2017/01/23. 10.4049/jimmunol.1601710 . 28115530

[21] Watson CT, Matsen FA, Jackson KJL, Bashir A, Smith ML, Glanville J, et al Comment on "A Database of Human Immune Receptor Alleles Recovered from Population Sequencing Data". J Immunol. 2017;198(9):3371–3. 10.4049/jimmunol.1700306 . 28416712

[22] Boyd SD, Gaëta BA, Jackson KJ, Fire AZ, Marshall EL, Merker JD, et al Individual variation in the germline Ig gene repertoire inferred from variable region gene rearrangements. J Immunol. 2010;184(12):6986–92. Epub 2010/05/21. 10.4049/jimmunol.1000445 20495067 PMC4281569

[23] Gadala-Maria D, Yaari G, Uduman M, Kleinstein SH. Automated analysis of high-throughput B-cell sequencing data reveals a high frequency of novel immunoglobulin V gene segment alleles. Proc Natl Acad Sci U S A. 2015;112(8):E862–70. Epub 2015/02/09. 10.1073/pnas.1417683112 25675496 PMC4345584

[24] Corcoran MM, Phad GE, Vázquez Bernat, Stahl-Hennig C, Sumida N, Persson MA, et al Production of individualized V gene databases reveals high levels of immunoglobulin genetic diversity. Nat Commun. 2016;7:13642 Epub 2016/12/20. 10.1038/ncomms13642 27995928 PMC5187446

[25] Zhang W, Wang IM, Wang C, Lin L, Chai X, Wu J, et al IMPre: An Accurate and Efficient Software for Prediction of T- and B-Cell Receptor Germline Genes and Alleles from Rearranged Repertoire Data. Front Immunol. 2016;7:457 Epub 2016/11/04. 10.3389/fimmu.2016.00457 27867380 PMC5095119

[26] Gadala-Maria D, Gidoni M, Marquez S, Vander Heiden JA, Kos JT, Watson CT, et al Identification of Subject-Specific Immunoglobulin Alleles From Expressed Repertoire Sequencing Data. Front Immunol. 2019;10:129 Epub 2019/02/13. 10.3389/fimmu.2019.00129 30814994 PMC6381938

[27] Khass M, Vale AM, Burrows PD, Schroeder HW. The sequences encoded by immunoglobulin diversity (D. Immunol Rev. 2018;284(1):106–19. 10.1111/imr.12669 . 29944758

[28] Safonova Y, Pevzner PA. Inference of Diversity Genes and Analysis of Non-canonical V(DD)J Recombination in Immunoglobulins. Front Immunol. 2019;10:987 Epub 2019/05/03. 10.3389/fimmu.2019.00987 31134072 PMC6516046

[29] Thörnqvist L, Ohlin M. Critical steps for computational inference of the 3'-end of novel alleles of immunoglobulin heavy chain variable genes—illustrated by an allele of IGHV3-7. Mol Immunol. 2018;103:1–6. Epub 2018/08/30. 10.1016/j.molimm.2018.08.018 . 30172112

[30] Ohlin M, Scheepers C, Corcoran M, Lees WD, Busse CE, Bagnara D, et al Inferred Allelic Variants of Immunoglobulin Receptor Genes: A System for Their Evaluation, Documentation, and Naming. Front Immunol. 2019;10:435 Epub 2019/03/18. 10.3389/fimmu.2019.00435 30936866 PMC6431624

[31] Mitzenmacher M. A survey of results for deletion channels and related synchronization channels. Probability Surveys. 2009;6:1–33.

[32] Levin M, Levander F, Palmason R, Greiff L, Ohlin M. Antibody-encoding repertoires of bone marrow and peripheral blood-a focus on IgE. J Allergy Clin Immunol. 2017;139(3):1026–30. Epub 2016/08/09. 10.1016/j.jaci.2016.06.040 . 27521279

[33] Ellebedy AH, Jackson KJ, Kissick HT, Nakaya HI, Davis CW, Roskin KM, et al Defining antigen-specific plasmablast and memory B cell subsets in human blood after viral infection or vaccination. Nat Immunol. 2016;17(10):1226–34. Epub 2016/08/15. 10.1038/ni.3533 27525369 PMC5054979

[34] Gupta NT, Adams KD, Briggs AW, Timberlake SC, Vigneault F, Kleinstein SH. Hierarchical Clustering Can Identify B Cell Clones with High Confidence in Ig Repertoire Sequencing Data. J Immunol. 2017;198(6):2489–99. Epub 2017/02/08. 10.4049/jimmunol.1601850 28179494 PMC5340603

[35] Friedensohn S, Lindner JM, Cornacchione V, Iazeolla M, Miho E, Zingg A, et al Synthetic Standards Combined With Error and Bias Correction Improve the Accuracy and Quantitative Resolution of Antibody Repertoire Sequencing in Human Naïve and Memory B Cells. Front Immunol. 2018;9:1401 Epub 2018/06/20. 10.3389/fimmu.2018.01401 29973938 PMC6019461

[36] Magri G, Comerma L, Pybus M, Sintes J, Lligé D, Segura-Garzón D, et al Human Secretory IgM Emerges from Plasma Cells Clonally Related to Gut Memory B Cells and Targets Highly Diverse Commensals. Immunity. 2017;47(1):118–34.e8. Epub 2017/07/11. 10.1016/j.immuni.2017.06.013 28709802 PMC5519504

[37] Stern JN, Yaari G, Vander Heiden JA, Church G, Donahue WF, Hintzen RQ, et al B cells populating the multiple sclerosis brain mature in the draining cervical lymph nodes. Sci Transl Med. 2014;6(248):248ra107 10.1126/scitranslmed.3008879 25100741 PMC4388137

[38] Greiff V, Menzel U, Miho E, Weber C, Riedel R, Cook S, et al Systems Analysis Reveals High Genetic and Antigen-Driven Predetermination of Antibody Repertoires throughout B Cell Development. Cell Rep. 2017;19(7):1467–78. 10.1016/j.celrep.2017.04.054 . 28514665

[39] Li X, Duan X, Yang K, Zhang W, Zhang C, Fu L, et al Comparative Analysis of Immune Repertoires between Bactrian Camel's Conventional and Heavy-Chain Antibodies. PLoS One. 2016;11(9):e0161801 Epub 2016/09/02. 10.1371/journal.pone.0161801 27588755 PMC5010241

[40] VanDuijn MM, Dekker LJ, van IJcken WFJ, Sillevis Smitt PAE, Luider TM. Immune Repertoire after Immunization As Seen by Next-Generation Sequencing and Proteomics. Front Immunol. 2017;8:1286 Epub 2017/10/16. 10.3389/fimmu.2017.01286 29085363 PMC5650670

[41] Banerjee S, Shi H, Banasik M, Moon H, Lees W, Qin Y, et al Evaluation of a novel multi-immunogen vaccine strategy for targeting 4E10/10E8 neutralizing epitopes on HIV-1 gp41 membrane proximal external region. Virology. 2017;505:113–26. Epub 2017/02/23. 10.1016/j.virol.2017.02.015 28237764 PMC5385849

[42] Shlemov A, Bankevich S, Bzikadze A, Turchaninova MA, Safonova Y, Pevzner PA. Reconstructing Antibody Repertoires from Error-Prone Immunosequencing Reads. J Immunol. 2017;199(9):3369–80. Epub 2017/10/04. 10.4049/jimmunol.1700485 28978691 PMC5661950

[43] Kidd MJ, Jackson KJ, Boyd SD, Collins AM. DJ Pairing during VDJ Recombination Shows Positional Biases That Vary among Individuals with Differing IGHD Locus Immunogenotypes. J Immunol. 2016;196(3):1158–64. Epub 2015/12/23. 10.4049/jimmunol.1501401 26700767 PMC4724508

[44] Kirik U, Greiff L, Levander F, Ohlin M. Parallel antibody germline gene and haplotype analyses support the validity of immunoglobulin germline gene inference and discovery. Mol Immunol. 2017;87:12–22. Epub 2017/04/04. 10.1016/j.molimm.2017.03.012 . 28388445

