# Supplementary material for: Automated analysis of immunosequencing datasets reveals novel immunoglobulin D genes across diverse species
Source: PLoS Comput Biol. 2020 Apr 27;16(4):e1007837. doi: 10.1371/journal.pcbi.1007837 (PMC7295240; doi:10.1371/journal.pcbi.1007837)
Supplement: S1 Notes — (PDF) [file pcbi.1007837.s001.pdf]

Supplemental Notes

Exact algorithm for solving the String Reconstruction Problem

Greedy Algorithm

MINING-D Parameters

Defining Relative Positions

Removing Unidirectional Extensions

Immunosequencing Datasets

Benchmarking MINING-D against IgScout

Novel Variations

Finding D genes in Whole Genome Sequencing Data

D Gene Usage

Overused D Genes

Highly Used D Genes in Non-human Datasets

Benchmarking MINING-D on simulated CDR3s

Benchmarking MINING-D on TCR datasets

Non-genomic insertions in naive and cord blood Rep-Seq datasets

Supplemental Note: Exact algorithm for solving the String Reconstruction Problem

It is easy to see that $P(C|s)$ is maximized by one of the modified strings. This observation leads to a brute-force algorithm for solving the String Reconstruction Problem (with complexity O($|s|$*N^2)) that simply computes $P(C|s)$ for each of the N modified strings. Below, we describe a O($|s|$*N) algorithm for solving this problem that is linear in the input size.

We assume for simplicity that all modified strings are different. This is not a strict assumption as one can always add special symbols to distinguish all strings. We denote $f(j) = \log(|\mathcal{A}|^{j+1} - 1)$ and search for a string that maximizes $\sum_{i=1}^{N} f(m_i)$. We denote a t-symbol prefix (*t-prefix*) of a string c as c^t and the set of all t-prefixes of strings from C as C^t. Given a string s and an integer t, we say that a string c is *t-similar* to s if t-prefixes of s and c coincide. The number of strings in C that are t-similar to s is denoted as $sim_t(C,s)$. Given a string s,

$$\text{score}(C^t|s^t) = \text{score}(C^{t-1}|s^{t-1}) + sim_t(C,s)\times \log\left(\frac{|\mathcal{A}|^{t+1} - 1}{|\mathcal{A}|^{t} - 1}\right). \quad (1)$$

We use this recurrence to efficiently compute score($C|s$) for each string s from C using dynamic programming. We construct a *trie* of all strings in C [1]. Each vertex in the trie is a t-prefix s^t of a string from C, and we recursively compute score ($C^t|s^t$) in each vertex of the trie using the above recurrence (assuming that the score of the root is $N\times\log(|\mathcal{A}| - 1)$). The optimal string is the string corresponding to the leaf node with the maximum score (Figure A). All scores can be computed by a single Depth First Search, assuming that all values $sim_t(C,s)$ are computed during the construction of the trie.

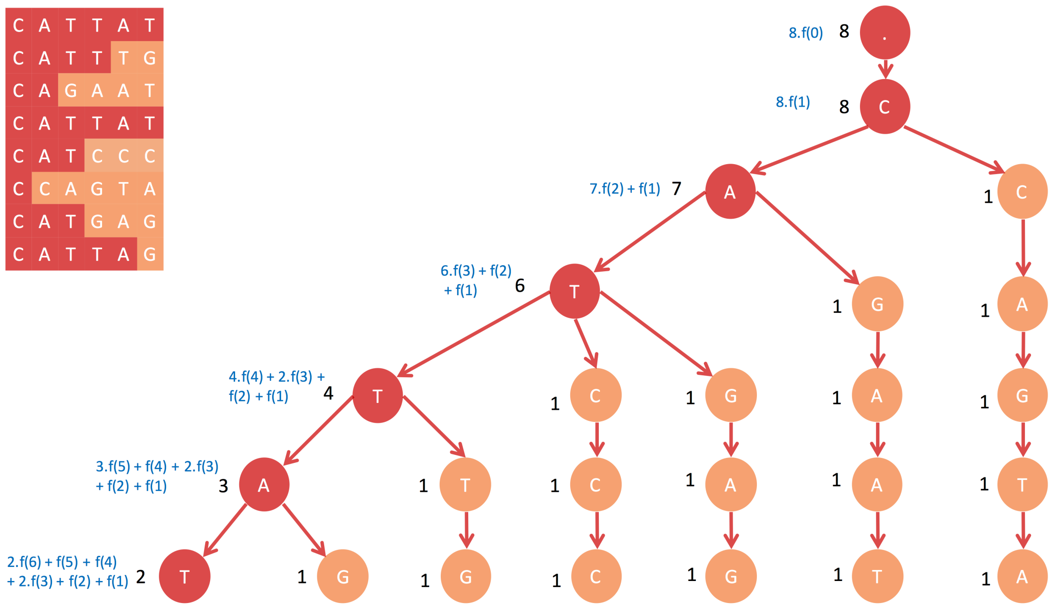

Figure A. Illustration of the algorithm for solving the String Reconstruction Problem. The set of modified strings is shown on the left, and their trie is shown on the right. The string associated with each vertex is the one that is formed by traversing from the root node to the vertex. The number of leaves under each vertex is shown on the left. The scores for all vertices in the path from the root node to the leaf node with the maximum score are shown in blue. The leaf CATTAT is the optimal seed string.

Supplemental Note: Greedy Algorithm

The pseudocode of the greedy algorithm is as follows:

greedy_string *(C):*
  $S_g \leftarrow emptystring()$
  $for\ j\ in\ 1\ to\ q:$
    $E(j) \leftarrow$ *most abundant symbol at position* j *in the strings in* C
    $S_g \leftarrow S_g + E(j)$
    $C \leftarrow$ *set of all strings* c_i *in* C *such that* $c_i[1{:}j] = S_g$
  $return\ S_g$

Figure B. Pseudocode of the greedy algorithm.

Supplemental Note: MINING-D Parameters

The most important parameter of the MINING-D algorithm is *m,* the number of seed *k-mers.* The default value of *m* should be different across species, since different numbers of D genes take part in the recombination process in each species. To decide on the default *m* for each species, we applied MINING-D to all datasets with different values of *m*. The results are shown in Table A. Based on the results in the table, we chose the following as the default values: human (*m* = 600), mouse (*m*= 300), rat (*m* = 300), rhesus macaque (*m* = 600), Bactrian camel (*m* = 300), and rabbit (*m* = 100).

The p-value threshold was chosen to be 10^{-36}. This value achieves 80% power from the test with a sample size of 2000 when the effect size (deviation from uniform distribution) is medium, according to the definition of the medium effect for chi-squared test. Having a strict (very low) threshold on the p-value may lead to some missing nucleotides on the sides of the genes, but since we are also doing genomic validation, the whole gene can be recovered from the genomic reads. On the other hand, high p-value threshold will not only lead to extra nucleotides on the sides, it will also cause more extensions to be made from a single *k*-mer, leading to more false positives. As another test, we also tried to extend the known human IMGT genes in Healthy Human CDR3 datasets using this threshold. 95% of the time, no extension was made to any gene.

Species - Individuals	IMGT Database	*m*	# Inferred genes including variations	# Inferred genes in IMGT	# Novel variations (validated)	# Novel genes (validated)
**Healthy Humans 20**	Human	1000	42	25	12 (2)	5 (0)
		600	38	25	8 (2)	5 (0)
		300	27	20	5 (2)	2 (0)
		100	15	12	3 (1)	-
**Untreated + Immunized Mouse 27**	Mouse	1000	35	18	12 (1)	5 (0)
		600	29	18	9 (1)	2 (0)
		300	24	18	5 (1)	1 (0)
		100	17	15	2 (1)	-
**Immunized Wistar Rat 1**	Rats	1000	27	13	11 (3)	4 (0)
		600	20	13	6 (3)	1 (0)
		300	16	12	4 (3)	-
		100	13	9	4 (3)	-
**Rhesus macaque - 7**	Crab-eating macaque	1000	25	17	6 (6)	2 (2)
		600	25	17	6 (6)	2 (2)
		300	24	16	6 (6)	2 (2)
		100	14	10	2 (2)	2 (2)
**Bactrian Camels 3**	Alpaca	1000	24	2	18 (8)	4 (0)
		600	19	2	15 (8)	2 (0)
		300	13	1	12 (8)	-
		100	10	1	9 (7)	-
**Immunized New Zealand Rabbit 1**	Rabbit	1000	73	3	57 (3)	13 (0)
		600	53	3	39 (3)	11 (0)

		300	34	3	25 (3)	6 (0)
		100	18	3	13 (3)	2 (0)

Table A. Information about inferred D genes. *m* denotes the number of seed 10-mers. The number of novel genes and variations validated using genomic data are also shown.

Supplemental Note: Defining Relative Positions

Looking at the relative positions of the extensions of *k*-mers has some advantages over looking at the relative positions of the *k*-mers. Since a relatively short *k*-mer can be a part of two of the three types of V, D, and J genes, the mean relative position among all the CDR3s of which such a *k*-mer is a substring can be misleading. Moreover, even if the *k*-mer is a substring of only one gene, the relative position of the extension gives a better estimate of the position of the CDR3 part of which the *k*-mer is a substring as illustrated in Figure C and Figure D.

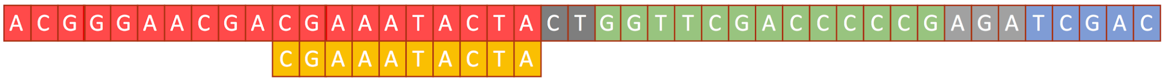

Figure C. The relative position of a 10-mer in a CDR3. The red, green, and blue colors represent parts of the V, D, and J segments in a CDR3 sequence. The relative position of the 10-mer CGAAATACTA is 0.32, whereas the relative position of its potential extension in red is 0.04.

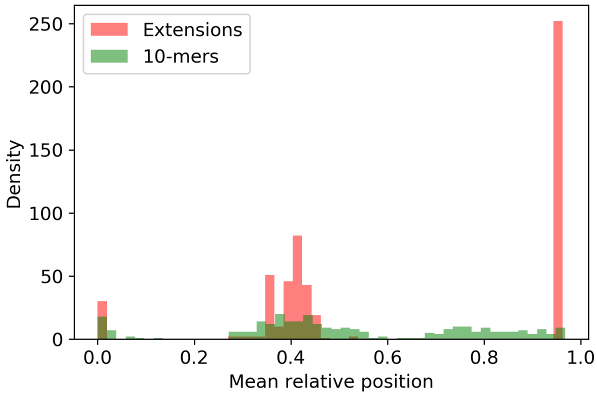

Figure D. The mean relative positions of the abundant seed 10-mers (in green) and their extensions (in red) in the MOUSE dataset. The relative positions of the extensions form three clusters, each corresponding to one of the V, D, and J genes.

Supplemental Note: Removing Unidirectional Extensions

Not all the unique extensions in the central cluster correspond to different D genes. Some of them are multiple reconstructions of the same D gene and are very similar to each other in the sense that they differ from each other by only a few nucleotides only at the edges. Most of them can be eliminated by making the observation that a highly abundant *k*-mer that the algorithm starts with might not always be, as a whole, a substring of a D gene. For example, the *k*-mer shown in Figure E can be among the highly abundant *k*-mers chosen to extend if the D gene shown in (a) is represented highly in the CDR3 sequences. When extended, it only extends to the right as shown in (c), retaining the random insertions in the *k*-mer. We can eliminate such *unidirectional* extensions because we expect some of the central *k*-mers of the D gene to also be among the highly abundant 10-mers. Such *k*-mers will be extended in both directions (*bidirectional* extensions), and by eliminating the unidirectional extensions, we reduce the number of reconstructions per D gene.

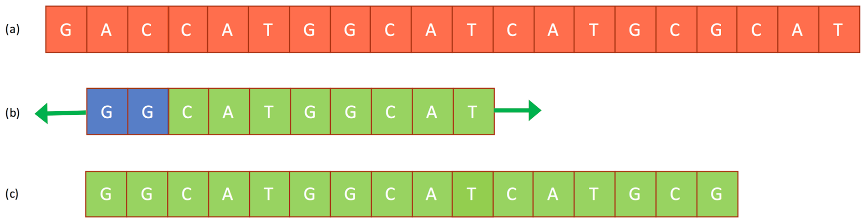

Figure E. A highly abundant 10-mer (b) that is formed by random insertions (two nucleotides in the beginning) and 8 nucleotides from a highly abundant D gene (a). Since this 10-mer was not a substring of the D gene, its extension (c) is also not a substring of the D gene.

Formally, let the number of nucleotides added to the left and right of the *k*-mer be N_L and N_R, respectively. We put the following constraint on N_L and N_R:

$$\frac{|N_L - N_R|}{\max(N_L, N_R)} \leq \alpha,$$

where α is a parameter of the algorithm. We used $\alpha = 0.5$. The possible values of N_L and N_R with $\alpha = 0.5$ are shown in Table B.

N_L	N_R
1	1,2
2	1,2,3,4
3	2,3,4,5,6
4	2,3,4,5,6,7,8
5	3,4,5,6,7,8,9,10

Table B. Possible values of N_L and N_R with the constraint when $\alpha = 0.5$.

Supplemental Note: Immunosequencing Datasets

Summaries of all the human and non-human immunosequencing datasets analyzed in this study are shown in Table C and Table D, respectively.

Name	# Individuals	Tissue	Cell Types	Isotypes	NCBI Project	# Datasets
Allergy Patients	6	PBMC, Bone Marrow	Unsorted	NA	PRJEB18926	24
Flu Vaccination	8	PBMC	Unsorted, Memory, resting memory, HA+/- memory, naive, ASC	NA	PRJNA324093	95
	3	PBMC	Unsorted	NA	PRJNA349143	18
Healthy	3	PBMC	Unsorted	IgG, IgM	PRJNA430091	28
Cord Blood	5	PBMC, Cord Blood	Unsorted	NA	PRJNA393446	6
Intestinal Repertoire	7	Ileum Mucosa, Colon Mucosa	Memory, Plasma	IgA, IgM	PRJNA355402	35

Multiple Sclerosis	4	Brain lesion, Cervical lymph node, Choroid plexus, Pia mater	Unsorted	NA	PRJNA248475	32
Hepatitis B (a)	9	PBMC	Unsorted, HBsAg+ and HLA-DR+ plasma cells	IgG	PRJNA308566	142
Hepatitis B (b)	9	PBMC	Unsorted, HBsAg+ and HLA-DR+ plasma cells	IgG, IgM	PRJNA308641	107

Table C. Summary of human immunosequencing datasets analyzed in the study. ASC refers to antibody secreting cells.

Species	Strains	Health Status	# Individuals	Tissue	Cell Types	Isotypes	Project	# Datasets
Mouse	C57BL/6J, Balb/c, Pet shop	Untreated, Antigen-immunized	27	Spleen, Bone marrow	pre-B cell, long lived plasma cell, naive B cell,	NA	PRJEB18631	71
Macaque	Rhesus macaques of Indian and Chinese origin	Healthy	7	PBMC	unsorted	IgM	PRJEB15295	7
Camel	Bactrian	Healthy	3	PBMC	PBMC	VH, VHH	PRJNA321369	6
Rat	Wistar	Immunized	10	Spleen	unsorted	NA	PRJNA386462	10
Rabbit	New Zealand white rabbit	Sequentially immunized	3	PBMC, Spleen	unsorted	NA	PRJNA355270	7

Table D. Summary of non-human immunosequencing datasets analyzed in the study.

Supplemental Note: Benchmarking MINING-D against IgScout

We compared the results of IgScout and MINING-D on all datasets from the project PRJEB18926. The results are shown in Figure F. A gene is said to be present in a dataset if at least one variation of the gene is found in the dataset and missing otherwise. In most datasets, both IgScout and MINING-D miss three D genes with very low usage (IGHD1-14, IGHD1-20, IGHD6-25) and a very short IGHD7-27 gene (11 nt). These D genes are also reported as missing in multiple studies on analyzing the usage of D genes [2-5]. While IgScout also misses three more short D genes with low usage i.e., IGHD1-1, IGHD4-4, IGHD1-7, MINING-D infers these genes for some individuals.

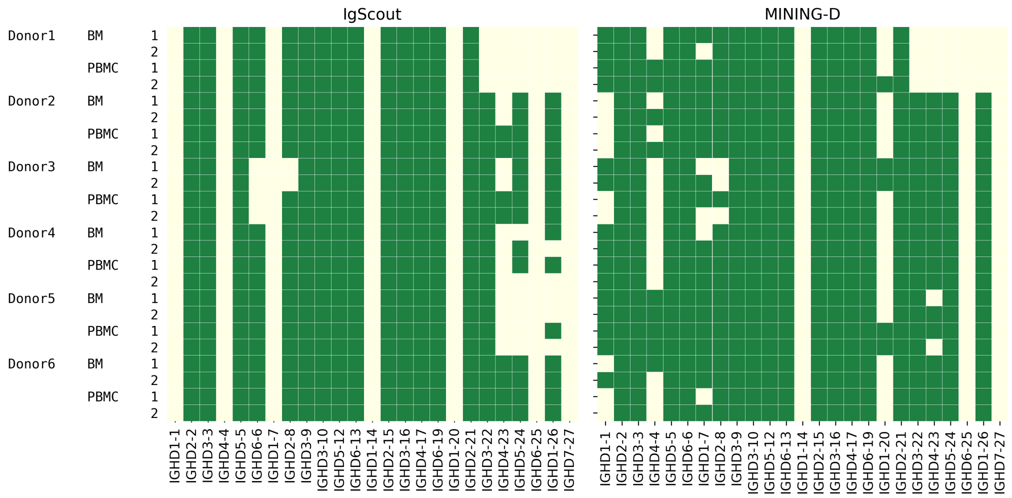

Figure F. Results of IgScout (left) and MINING-D (right) on datasets from the project PRJEB18926. All genes that were found in a dataset are shown in dark green, whereas the missing genes in datasets are denoted by light green. Missing inferences for genes IGHD3-22 through IGHD7-27 in Donor 1 indicate a potential deletion polymorphism in the IGHD locus.

We also compared the MINING-D and IgScout results on non-human datasets. Tables E-G compare the results of IgScout and MINING-D on ten Mouse datasets (4 Balb/c mice, 4 C57BL/6 mice, and 2 pets), all Rat datasets, and all Camel datasets. Figure G presents the distributions of missing and extra nucleotide bases in the inferred genes (as compared to the IMGT genes for all mouse datasets) for both MINING-D and IgScout.

**Strain**	**Dataset**	**Inferred IMGT D genes**		
		**Both**	**IgScout only**	**MINING-D only**
Balb/c	ERR1759659	IGHD1-1*0<br>IGHD1-2*01<br>IGHD2-1*01<br>IGHD2-14*01<br>IGHD2-3*01<br>IGHD2-4*01<br>IGHD3-2*01<br>IGHD4-1*01<br>IGHD2-10*01	-	IGHD2-10*02<br>IGHD2-2*01
Balb/c	ERR1759660	IGHD1-1*01<br>IGHD1-2*01<br>IGHD2-14*01<br>IGHD2-3*01<br>IGHD2-4*01<br>IGHD3-2*01<br>IGHD4-1*01<br>IGHD2-10*01	-	IGHD2-10*02<br>IGHD2-2*01
Balb/c	ERR1759661	IGHD1-1*01<br>IGHD1-2*01<br>IGHD2-14*01<br>IGHD2-3*01<br>IGHD2-4*01<br>IGHD3-2*01<br>IGHD4-1*01	-	IGHD2-10*02<br>IGHD2-2*01

		IGHD2-10*01		
Balb/c	ERR1759662	IGHD1-1*01<br>IGHD1-2*01<br>IGHD2-1*01<br>IGHD2-14*01<br>IGHD2-3*01<br>IGHD2-4*01<br>IGHD3-2*01<br>IGHD4-1*01	-	IGHD2-10*02<br>IGHD2-2*01
C57BL/6	ERR1759665	IGHD1-1*01<br>IGHD2-1*01<br>IGHD2-3*01<br>IGHD2-4*01<br>IGHD2-5*01<br>IGHD3-2*02<br>IGHD4-1*01	-	IGHD2-2*01
C57BL/6	ERR1759668	IGHD1-1*01<br>IGHD2-1*01<br>IGHD2-3*01<br>IGHD2-4*01<br>IGHD2-5*01<br>IGHD4-1*01<br>IGHD3-2*02	-	IGHD2-2*01
C57BL/6	ERR1759671	IGHD1-1*01<br>IGHD2-1*01<br>IGHD2-3*01<br>IGHD2-4*01<br>IGHD2-5*01<br>IGHD3-2*02<br>IGHD4-1*01	-	IGHD2-2*01
C57BL/6	ERR1759674	IGHD1-1*01<br>IGHD2-1*01<br>IGHD2-3*01<br>IGHD2-4*01<br>IGHD2-5*01<br>IGHD4-1*01<br>IGHD3-2*02	-	IGHD2-2*01
Pet	ERR1759679	IGHD1-1*01<br>IGHD1-2*01<br>IGHD2-14*01<br>IGHD2-3*01<br>IGHD2-4*01<br>IGHD3-2*02<br>IGHD4-1*01	IGHD2-10*01	IGHD2-1*01<br>IGHD2-2*01<br>IGHD2-5*01<br>IGHD3-2*01
Pet	ERR1759680	IGHD1-1*01<br>IGHD1-2*01<br>IGHD2-14*01<br>IGHD2-3*01<br>IGHD2-4*01<br>IGHD3-2*01<br>IGHD4-1*01<br>IGHD2-10*01	-	IGHD2-2*01

Table E. Comparison of IMGT genes inferred by IgScout and MINING-D in various Mouse datasets. The gene IGHD2-10*01 (the only gene inferred by IgScout but missed by MINING-D) and the gene IGHD2-1*01 (inferred by MINING-D but missed by IgScout) only differ at the first position.

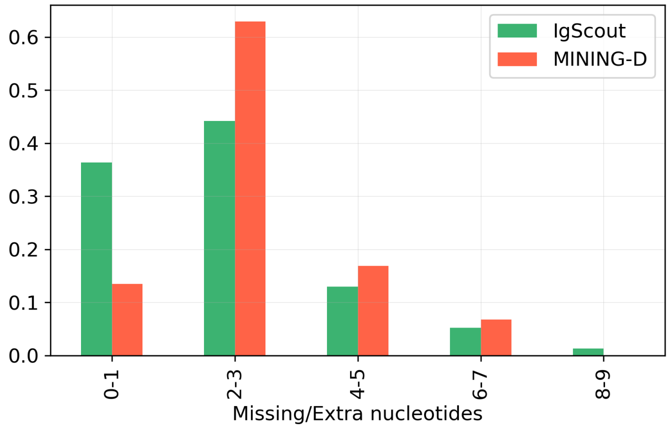

Figure G. Distribution of missing or extra nucleotide bases in the inferred genes as compared to the IMGT genes for all mouse datasets shown in Table E. Only genes that were inferred by both IgScout and MINING-D were included in the comparison.

**Dataset**	**Inferred IMGT D genes**		
	**Both**	**IgScout only**	**MINING-D only**
SRR5534359	IGHD1-10*01<br>IGHD1-11*01<br>IGHD1-12*03<br>IGHD1-2*01<br>IGHD1-4*01<br>IGHD1-5*01<br>IGHD1-6*01<br>IGHD4-3*01<br>IGHD5-1*01	-	IGHD1-1*01<br>IGHD1-12*02
SRR5534360	IGHD1-1*01<br>IGHD1-10*01<br>IGHD1-11*01<br>IGHD1-2*01<br>IGHD1-4*01<br>IGHD1-5*01<br>IGHD1-6*01<br>IGHD1-9*01<br>IGHD4-3*01	IGHD5-1*01	IGHD1-12*02<br>IGHD1-12*03<br>IGHD1-8*01
SRR5534361	IGHD1-11*01<br>IGHD1-2*01<br>IGHD1-6*01<br>IGHD4-3*01	-	IGHD1-1*01<br>IGHD1-10*01<br>IGHD1-12*02<br>IGHD1-12*03<br>IGHD1-4*01<br>IGHD1-5*01
SRR5534362	IGHD1-1*01<br>IGHD1-10*01<br>IGHD1-11*01<br>IGHD1-2*01	IGHD5-1*01	IGHD1-12*02<br>IGHD1-12*03

	IGHD1-4*01<br>IGHD1-5*01<br>IGHD1-6*01<br>IGHD4-3*01		
SRR5534363	IGHD1-10*01<br>IGHD1-11*01<br>IGHD1-2*01<br>IGHD1-4*01<br>IGHD1-5*01<br>IGHD1-6*01<br>IGHD4-3*01<br>IGHD5-1*01	-	IGHD1-1*01<br>IGHD1-12*02<br>IGHD1-12*03
SRR5534364	IGHD1-1*01<br>IGHD1-10*01<br>IGHD1-11*01<br>IGHD1-12*02<br>IGHD1-2*01<br>IGHD1-4*01<br>IGHD1-5*01<br>IGHD1-6*01<br>IGHD1-9*01<br>IGHD4-3*01<br>IGHD5-1*01	-	IGHD1-12*03<br>IGHD1-8*01
SRR5534365	IGHD1-1*01<br>IGHD1-10*01<br>IGHD1-11*01<br>IGHD1-2*01<br>IGHD1-4*01<br>IGHD1-5*01<br>IGHD1-6*01<br>IGHD1-9*01<br>IGHD4-3*01<br>IGHD5-1*01	-	IGHD1-12*02<br>IGHD1-12*03<br>IGHD1-7*01<br>IGHD1-8*01
SRR5534366	IGHD1-10*01<br>IGHD1-11*01<br>IGHD1-12*02<br>IGHD1-12*03<br>IGHD1-2*01<br>IGHD1-4*01<br>IGHD1-5*01<br>IGHD1-6*01<br>IGHD1-8*01<br>IGHD1-9*01<br>IGHD4-3*01<br>IGHD5-1*01	-	IGHD1-1*01
SRR5534367	IGHD1-10*01<br>IGHD1-11*01<br>IGHD1-12*02<br>IGHD1-2*01<br>IGHD1-4*01<br>IGHD1-5*01<br>IGHD1-6*01<br>IGHD1-8*01<br>IGHD1-9*01	IGHD5-1*01	IGHD1-1*01<br>IGHD1-12*03

	IGHD4-3*01		
SRR5534368	IGHD1-1*01<br>IGHD1-10*01<br>IGHD1-11*01<br>IGHD1-4*01<br>IGHD1-5*01<br>IGHD1-6*01<br>IGHD1-9*01<br>IGHD4-3*01<br>IGHD5-1*01	-	IGHD1-12*02<br>IGHD1-12*03<br>IGHD1-2*01<br>IGHD1-8*01

Table F. Comparison of IMGT genes inferred by IgScout and MINING-D from various Rat datasets.

Gene (Alpaca)	Variant	1 VH	1 VHH	2 VH	2 VHH	3 VH	3 VHH
IGHD6*01	N_Var (IGHD6*01) - 2	M-I	M-I	M-I	M-I	M-I	M-I
IGHD2*01	N_Var (IGHD2*01) - 1	M-I	M-I	-	M-I	M-I	M-I
	N_Var (IGHD2*01) - 0	-	-	M-I	-	-	-
IGHD3*01	N_Var (IGHD3*01) - 1	M-I	M-I	M	M-I	M-I	M-I
	N_Var (IGHD3*01) - 0	-	-	M-I	-	-	-
IGHD4*01	N_Var (IGHD4*01) - 1	M-I	M-I	M	-	M	-
	N_Var (IGHD4*01) - 0	-	-	M-I	-	-	-
IGHD5*01	N_Var (IGHD5*01) - 0	M	M	-	M	M	M
	IGHD5*01	-	-	M	-	-	-

Table G. Comparison of genes inferred by IgScout and MINING-D from the Camel datasets. M-I denotes that a gene was inferred by both MINING-D and IgScout, whereas M denotes that the gene was inferred by MINING-D only. IMGT genes in this table refer to the IMGT alpaca genes. Only genes that were validated using genomic reads are included in this comparison.

Supplemental Note: Novel Variations

All the variations found using MINING-D for humans, camels, rhesus macaques, mice, rats, and rabbits are shown in Table H. The polymorphisms in the genes validated using genomic data are highlighted in red.

Human

```
IGHD3-10*01
Original     GTATTACTATGGTTCGGGGAGTTATTATAAC
N_Var-3      GTATTACTATGGTTCAGGGAGTTATTATAAC
N_Var-2      --------ATGGTTCGGGGAcTTAT------
N_Var-1      ---------TGGTTCGGGGAaTTA-------
```

```
IGHD3-22*01
Original     GTATTACTATGATAGTAGTGGTTATTACTAC
N_Var-1      --ATTACTATGATAcTAGTGG----------
N_Var-0      -------TATGATAGcAGTGGT---------
```

```
N_Var-0      ---------TGaTTCGGGGAGTT--------
```

```
IGHD2-2*01
Original     AGGATATTGTAGTAGTACCAGCTGCTATGCC
N_Var-0      AGGATATTGTAtTAGTACCAGCTGCTAT---
```

```
IGHD3-16*02
Original     GTATTATGATTACGTTTGGGGGAGTTATCGTTATACC
N_Var-0      ---TTATGATTACATTTGGGGGAGTTATCGTTAT---
```

Camel

```
IGHD3*01 (Alpaca)
Original     GTATTACTACTGCTCAGGCTATGGGTGTTATGAC
N_Var-1      ----GACTGCTATTCAGGCTCTTGGTGTTATG--
N_Var-0      ---TGACTACTGTTCAGGCTCTTGGTGT------
```

```
IGHD2*01 (Alpaca)
Original     ACATACTATAGTGGTAGTTACTACTACACC
N_Var-1      --ATATTGTAGTGGTGGTTACTGCTAC---
N_Var-0      -CATACTATAGTGGTGGTTACTAC------
```

```
IGHD4*01 (Alpaca)
Original     TTACTATAGCGACTATGAC
N_Var-1      CTACTATAGCGACTATG--
N_Var-0      -TACTATAACGAATATG--
```

```
IGHD6*01 (Alpaca)
Original     GTACGGTAGTAGCTGGTAC----
N_Var-4      --ACGGTgGTAGtTGGT------
N_Var-3      ---CGGTgGTAGgTGGTggctgg
N_Var-2      GTACGGTGGTAGCTGGTAC----
N_Var-1      ---CGGTgGTAcCTGGT------
N_Var-0      --ACGGTgGTAaCTGG-------
```

```
IGHD5*01 (Alpaca)
Original     AGACTACGGGTTGGGGTAC
N_Var-0      ----TATGGGTT-GGGTAC
```

Rhesus Macaque

```
IGHD1S39*01
Original     GGTATAGTGGGAACTACAAC
N_Var-0      -----AGTGGGAGCTAC---
```

```
IGHD3S18*01
Original     GTACTGGGGTGATTATTATGAC
N_Var-0      --ACTGGAGTGATTATTA----
```

```
IGHD5S3*01
Original     GTGGATACAGTGGGTACAGTTAC
N_Var-0      ---GATACAGCGGGTACAGT---
```

```
IGHD2S11*01
Original     AGAATATTGTAGTAGTACTTACTGCTCCTCC
N_Var-0      -----ATTGTAGTGGTACTTACTGCT-----
```

```
IGHD2S17*01
Original     AGAATACTGTACTGGTAGTGGTTGCTATGCC
N_Var-0      ----TACTGTACTGGTAGTGGTTGCTAC---
```

```
IGHD3S23*01
Original     GTATTACTATGATAGTGGTTATTACACCCACAGCGT
N_Var-0      ---TTACTATGGTAGTGGTTATTAC-----------
```

Mouse

```
IGHD1-1*01
Original     TTTATTACTACGGTAGTAGCTAC-
N_Var-3      ------ACgACGGTAGTAGC----
N_Var-2      -TTATTACTACGGTAGTAGagggg
N_Var-1      ---ATTACTgCGGTAGTAGCTAC-
N_Var-0      TTTATTACTACGATGGTAGCTACg
```

```
IGHD2-4*01
Original     TCTACTATGATTACGAC---
N_Var-0      ------ggGATTACGACagg
```

Rat

```
IGHD1-3*01
Original     TTTTTAACTATGGTAGCTAC
N_Var-0      -TTTTAACTACGGTAGCTAC
```

```
IGHD1-9*01
Original     TACATACTATGGGTATAACTAC-
N_Var-1      --CATACTACGGGTATACCTACg
N_Var-0      --CATACTAcGGGTATAACTAC-
```

```
IGHD1-12*02
Original     TTTATTACTATGATGGTAGTTATTACTAC-
N_Var-0      -TTATTACTATGATGGTACTTATTACTACg
```

Rabbit

```
IGHD6-1*01
Original     --------------GTTACTATAGTTATGGTTATGCTTATGCTACC
N_Var-7      ---------------TTAtgATgGTTATGGTTATGgTa--------
N_Var-6      tta------tagtgGTTAtggTgGTTATGcTTATG-----------
N_Var-5      ---------------TggtTATgGTgATGGTTATGCT---------
N_Var-4      TTACTATACTTATGGTTATGCTGGTTATGCTTATGCTAC-------
N_Var-3      TTA------TGCTGGTTATGCTGGTTATGGTTATGCTAC-------
N_Var-2      ------------tgGTTAtggTgGTTATGGTTATG-----------
N_Var-1      --------------------ATAcTTATGGTTATGgTggT------
N_Var-0      --------------------ATAGTTATGGTTATGgTg--------
```

```
IGHD1-1*01
Original     GCATATACTAGTAGTAGTGGTTATTATATAC
N_Var-2      GCATATGCTAGTAGTAGTGGTTATTAT----
N_Var-1      -----------TgGTAGTGGTTATTAT----
N_Var-0      -------------GTAGTGGTggTTAT----
```

```
IGHD2-1*01
Original     TAGCTACGATGACTATGGTGATTAC-
N_Var-0      ---------TGAtTATGGTGgTTAtg
```

```
IGHD8-1*01
Original     GTTATGCTGGTAGTAGTTATTATACC
N_Var-0      -TTATGCTGGTgaTgGTTATg-----
```


Table H. All Inferred novel variations. Variations that were validated using genomic data are shown by highlighting the polymorphisms in red.

Supplemental Note: Finding D genes in Whole Genome Sequencing data

Tables I-M show the number of WGS reads confirming both novel and known variations of D genes and demonstrate that novel and known D genes have similar numbers of supporting reads in the selected WGS datasets.

**Dataset**	**IGHD3-10**			**IGHD3-16**			**IGHD3-22**
	***01**	***02**	**N_Var (IGHD3-10*01)-3**	***01**	***02**	**N_Var (IGHD3-16*02)-0**	
SRR6435661	8	-	14	-	42	30	16
SRR6435676	19	-	12	-	77	58	28
SRR6435686	14	-	8	-	20	32	12
SRR6435691	4	-	12	-	35	32	13
SRR6435692	6	-	12	-	35	36	15

Table I. Number of genomic reads containing exact occurrences of known and novel allelic variants of human genes IGHD3-10, IGHD3-16, and IGHD3-22 in five datasets containing reads with the novel allelic variants. Information on the number of datasets that a gene is present in and the number of reads containing the gene for some other genes - IGHD2-2*01: 36(4-54), IGHD3-3*01: 30(6-45), IGHD3-22*01: 40(5-29).

**Gene**	**Type**	**SRR7865780**	**SRR7865781**	**SRR7865793**	**SRR7865795**
IGHD1S39	IMGT	26	16	11	0
	Novel	0	0	0	18
IGHD3S18	IMGT	69	45	54	29
	Novel	0	21	0	8
IGHD5-S3	IMGT	74	58	73	41
	Novel	28	22	22	13
IGHD2S11	IMGT	24	27	36	7
	Novel	0	0	0	6
IGHD2S17	IMGT	0	0	0	9

	Novel	30	12	32	8
IGHD3S23	IMGT	0	9	8	11
	Novel	24	12	17	0
N_Gene-0		27	9	21	9
N_Gene-1		0	8	0	9

Table J. Number of genomic reads containing exact occurrences of known and novel allelic variants of macaque genes. IGHD3S18*01 has the same sequence as IGHD3S29*01 that results in higher coverage than the novel allelic variant. Similarly, the coverage for IGHD5S3*01 is higher than the novel variant because it has the same sequence as IGHD5S25*01.

**Gene**	**IGHD1-3**		**IGHD1-9**		**IGHD1-12**			
	IMGT	Novel	IMGT	Novel	IMGT*01	IMGT*02	IMGT*03	Novel
SRR7503107	5	11	-	2	-	-	4	8
SRR7503108	8	6	-	4	-	-	2	1
SRR7503109	8	7	-	5	-	-	4	8
SRR7503110	12	7	-	4	-	-	6	8
SRR7503111	5	6	-	5	-	-	4	9
SRR7503112	6	2	-	2	-	-	7	2
SRR7503113	7	5	-	3	-	-	4	7
SRR7503114	12	4	-	6	-	-	7	6
SRR7503115	9	18	-	8	-	-	17	9
SRR7503116	1	5	-	5	-	-	2	3

Table K. Number of genomic reads containing exact occurrences of known and novel allelic variants of rat genes.

**Gene**	**Type**	**Datasets present in (#Reads)**
IGHD6-1	IMGT	8 (1-5)
	N_Var (IGHD6-1*01)-3	19 (1-9)
	N_Var (IGHD6-1*01)-4	11 (1-6)
IGHD1-1	IMGT	6 (2-3)

	N_Var (IGHD1-1*01)-2	23 (1-14)

Table L. Number of datasets that have a genomic read containing the exact occurrence of a gene and the number of reads containing exact occurrences for known and novel allelic variants of rabbit genes.

Gene	Type	SRR1947239	SRR1947240	SRR1947241	SRR1947242	SRR1947243	SRR1947244	SRR1947245
IGHD3*01	IMGT	-	-	-	-	-	-	-
	N_Var - 0	-	-	-	-	2	6	-
	N_Var - 1	16	6	7	12	1	8	11
IGHD2*01	IMGT	-	-	-	-	-	-	-
	N_Var - 0	-	-	-	-	2	2	-
	N_Var - 1	10	11	5	17	4	-	5
IGHD4*01	IMGT	-	-	-	-	-	-	-
	N_Var - 0	-	-	-	-	5	4	-
	N_Var - 1	14	11	15	10	-	7	10
IGHD5*01	IMGT	-	-	-	-	-	-	-
	N_Var - 0	13	9	2	6	5	0	3
IGHD6*01	IMGT	-	-	-	-	-	-	-
	N_Var - 2	11	9	21	11	5	14	14

Table M. Number of genomic reads containing exact occurrences of inferred camel genes. The IMGT genes here correspond to alpaca genes.

Supplemental Note: D Gene Usage

Usage of D genes in the Flu Vaccination dataset. To analyze the usage of the D genes in different types of cells (including hemagglutinin-positive (HA+) and HA- activated B cells, antibody secreting cells, memory cells, and naive cells) from PBMCs at different times after flu vaccination, we used 95 datasets from the NCBI project PRJNA324093. 55.3% of CDR3s were traceable on average in all the datasets. The D gene usage profiles are very different in HA+ cells and other cells for almost all individuals suggesting the usage of specific D genes for HA+ clones in those individuals (Figure H). Interestingly, the overused D genes are not the same across individuals. For instance, for individual 7, genes IGHD2-21*02 and IGHD4-17*01 are overused, and for individual 6, only the gene IGHD4-17*01 is overused.

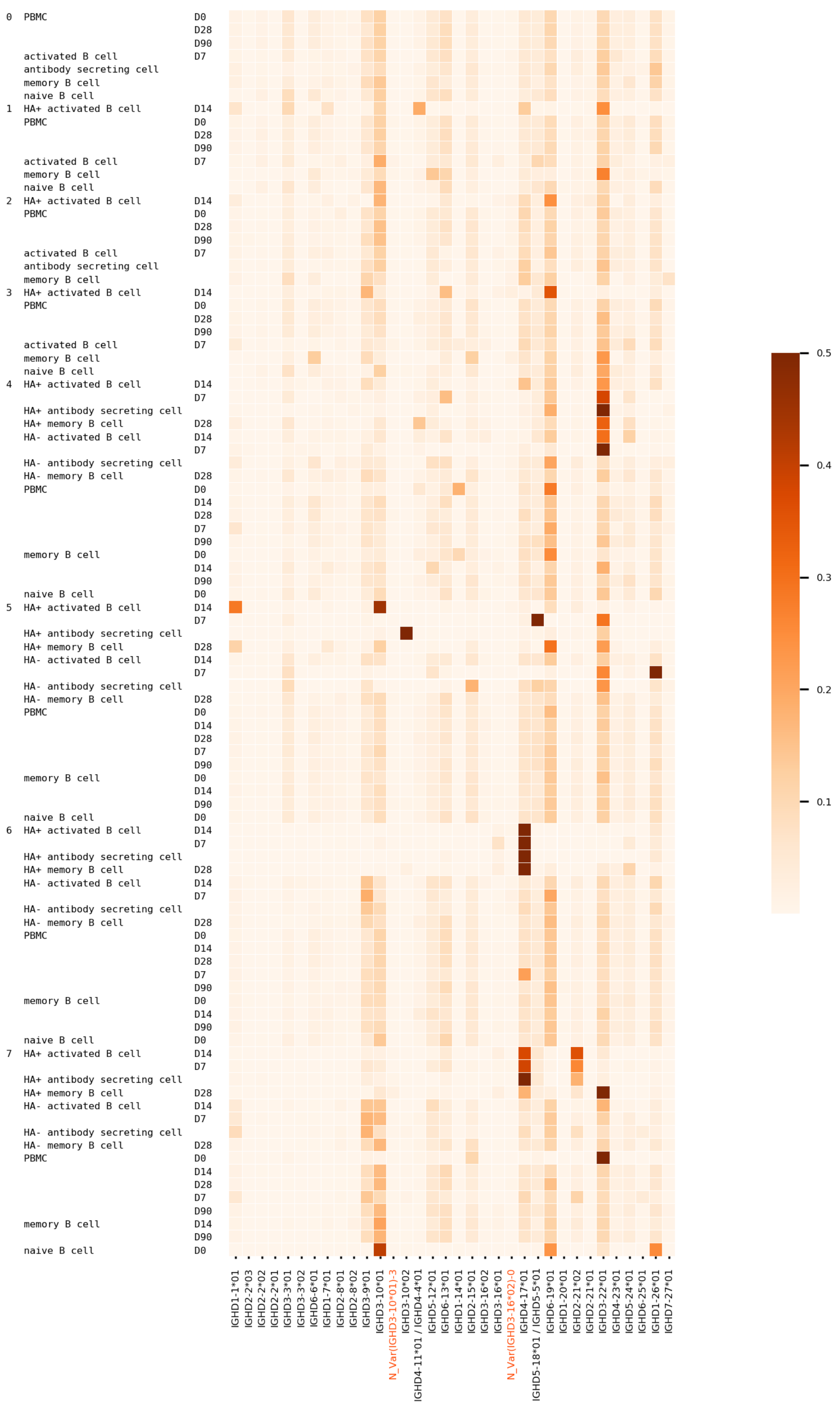

Figure H. Usage of IMGT and novel variations of IGHD genes in various datasets corresponding to flu vaccination. The columns on the left represent the individual, the cell type, and the time point (day after vaccination).

Usage of D genes in the Multiple Sclerosis dataset. 45.2% of CDR3s on average were traceable in each dataset. The usage of D genes across datasets from tissues such as brain lesion, cervical lymph node, choroid plexus, and pia mater is shown in Figure I. The results suggest that the usage of genes is different in different tissues from the same individual. For instance, for individual M5, IGHD1-26*01

and IGHD3-3*01 are overused in choroid plexus, whereas only IGHD3-3*01 is overused in brain lesion compared to other tissues.

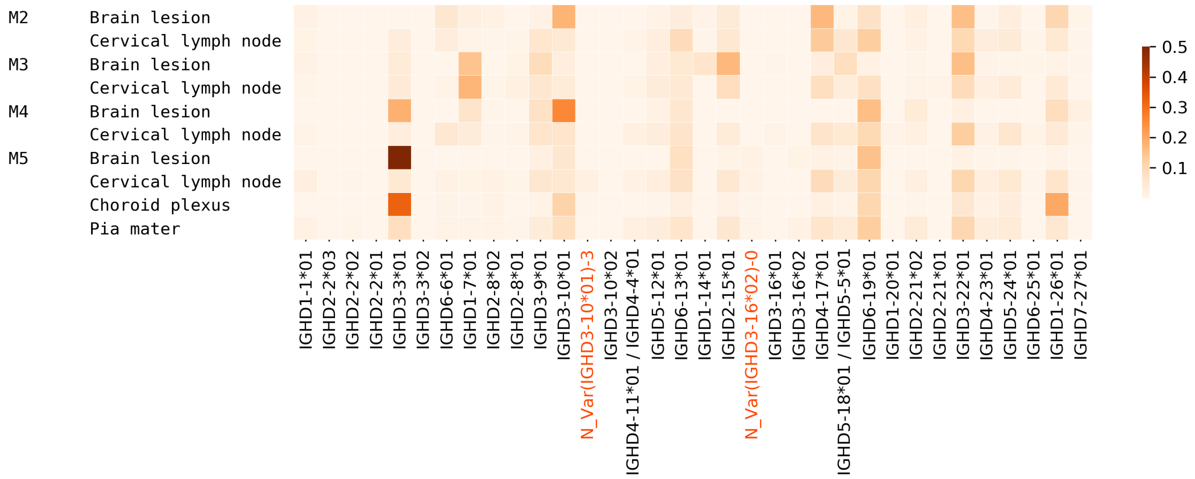

Figure I. Usage of various known and novel genes in various datasets corresponding to different tissues in Multiple Sclerosis patients. The columns on the left represent the individual and the tissue, respectively.

Usage of D genes in the Intestinal Repertoire dataset. We analyzed the usage of D genes in datasets corresponding to memory and plasma cells, IgA and IgM isotypes from ileum and colon tissues from 4 individuals, and naive cells from ileum from 3 individuals (Figure J). 43.5% of CDR3s on average were traceable in each dataset. For IgM naive cells (ileum mucosa), the number of traceable CDR3s was 71.42% on average, whereas for memory and plasma cells from the same tissue, it was 43.25% and 43.12%, respectively. The D gene IGHD3-3*01 was used significantly less in plasma and memory cells from both tissues compared to naive cells from ileum and PBMCs from healthy individuals (Figure 4). Similarly, the gene IGHD6-6*01 seems to be under-used in plasma and memory cells from the ileum tissue compared to naive cells. Subtle differences can also be found among the usage between different isotypes from the same individual's tissue, e.g., genes IGHD2-21*02, IGHD2-8*01, IGHD3-16*02, IGHD5-5*01/IGHD5-18*01, and IGHD7-27*01 are presented more in the IgM isotype than the IgA isotype in the colon tissue from individual 0.

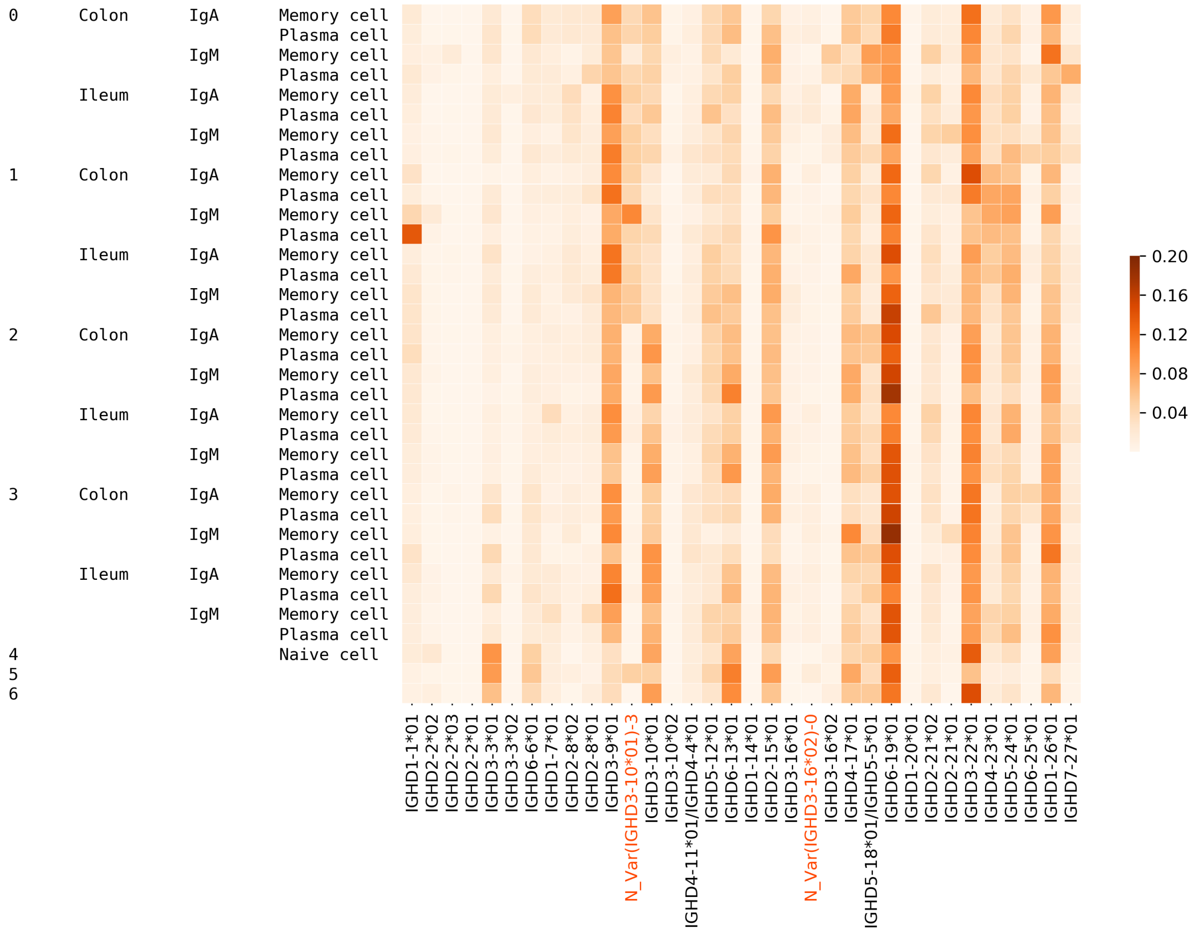

Figure J. Usage of various known and novel genes in various datasets corresponding to human intestinal antibodies. The columns on the left represent the individual, tissue, isotype, and cell type, respectively.

Usage of D genes in the Hepatitis B Vaccination dataset. To study the usage of D genes in HbsAg+ B cells and HLA-DR+ plasma cells, we analyzed datasets corresponding to individuals who received a Hepatitis B vaccination. 51.3% of CDR3s on average were traceable in each dataset. IgM and IgG datasets had 65.4% and 45.9% traceable CDR3s on average, respectively. The usage of genes is shown in Figure K. Differences in the usage profiles can be seen among HbsAg+ B cells, HLA-DR+ plasma cells, and PBMCs from the same individual for most of the individuals. For instance, for individual 7, IGHD2-15*01 is under-used in both HbsAg+ B cells and HLA-DR+ plasma cells compared to PBMCs, whereas genes IGHD4-17*01 and IGHD4-23*01 are overused. The gene IGHD3-22*01 is unpresented and the genes IGHD5-5*01/IGHD5-18*01 and IGHD7-27*01 are overused in HLA-DR+ plasma cells compared to HbsAg+ B cells. For individual 2, as another example, the genes IGHD3-22*01, IGHD3-3*01, and IGHD6-13*01 do not appear to be presented in the CDR3s from HLA-DR+ plasma cells, although they are presented in both the PBMCs and HbsAg+ B cells from the same individual. Similarly, differences between profiles can be found for all individuals, although there does not appear to be a strong pattern across individuals, suggesting that the response is highly personalized and might depend upon other factors.

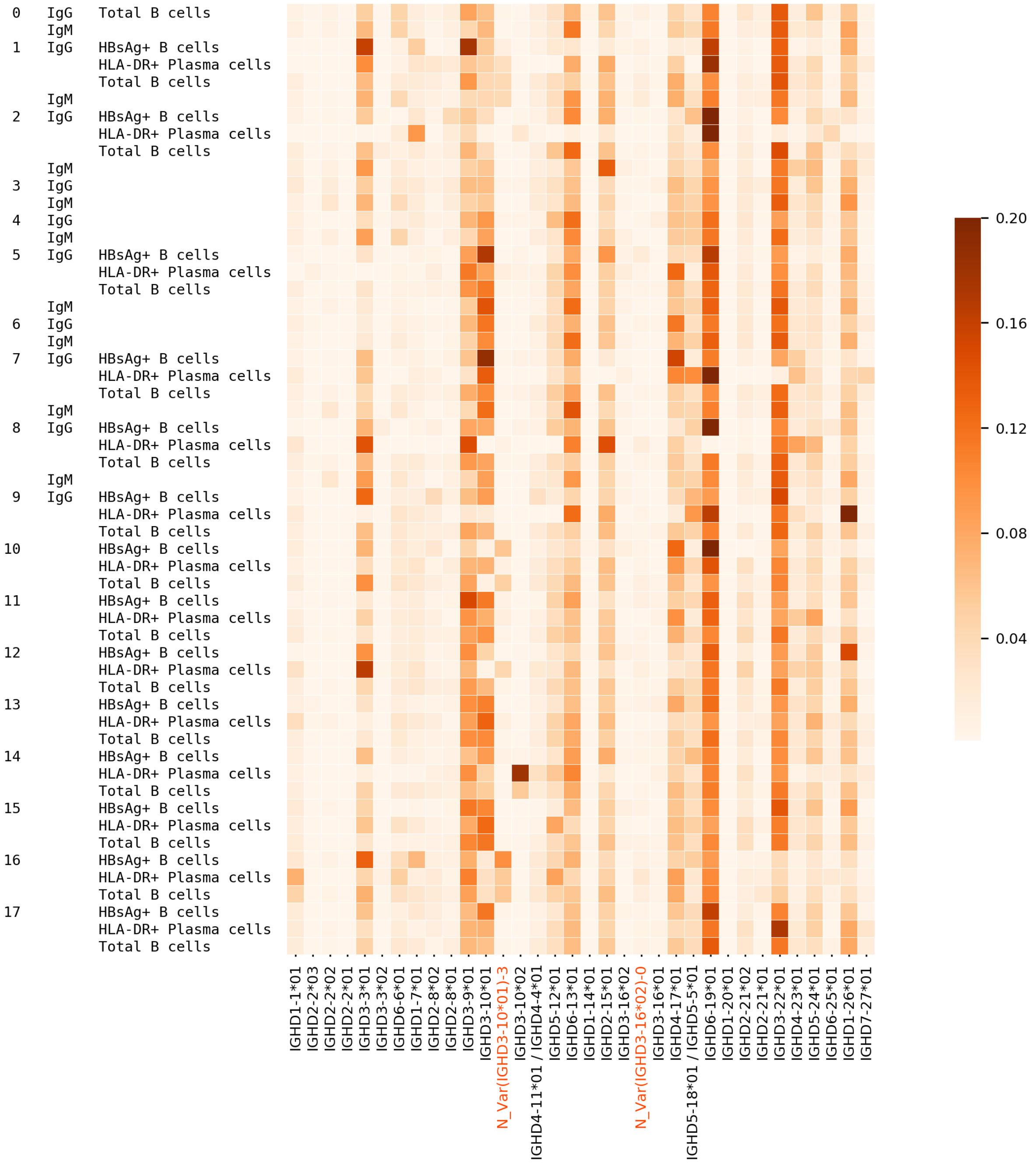

Figure K. Usage of various known and novel genes in different datasets corresponding to different cell types and isotypes corresponding to human subjects with hepatitis B vaccination.

Usage of D genes in Cord Blood dataset. 48.9% of CDR3s were traceable on average in the PBMC datasets, whereas 71.6% of the datasets were traceable in the Cord Blood datasets (Figure L). Supplemental Note: "Non-genomic insertions in naive and cord blood Rep-Seq datasets" shows that the Cord Blood datasets are characterized by smaller number of VD and DJ insertions compared to the naïve datasets.

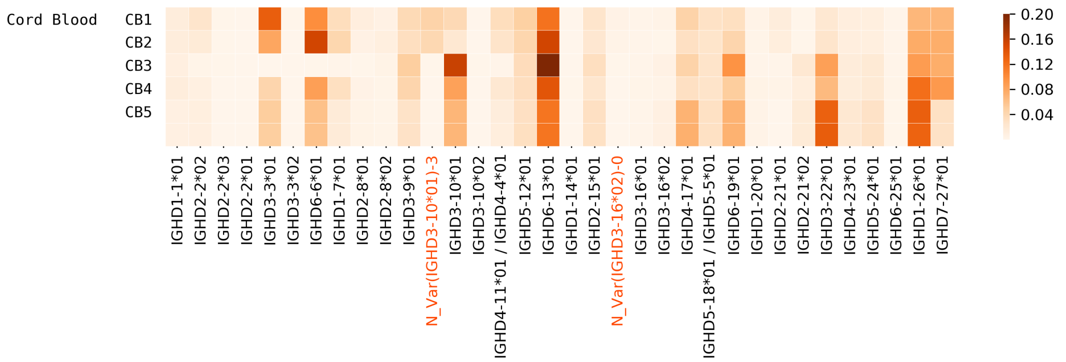

Figure L. Usage of various known and novel genes in cord blood datasets.

Usage of D genes in mice datasets.

Figure M shows usage of various known and novel genes/variations in different datasets corresponding to different strain, cell type, and tissue from mice.

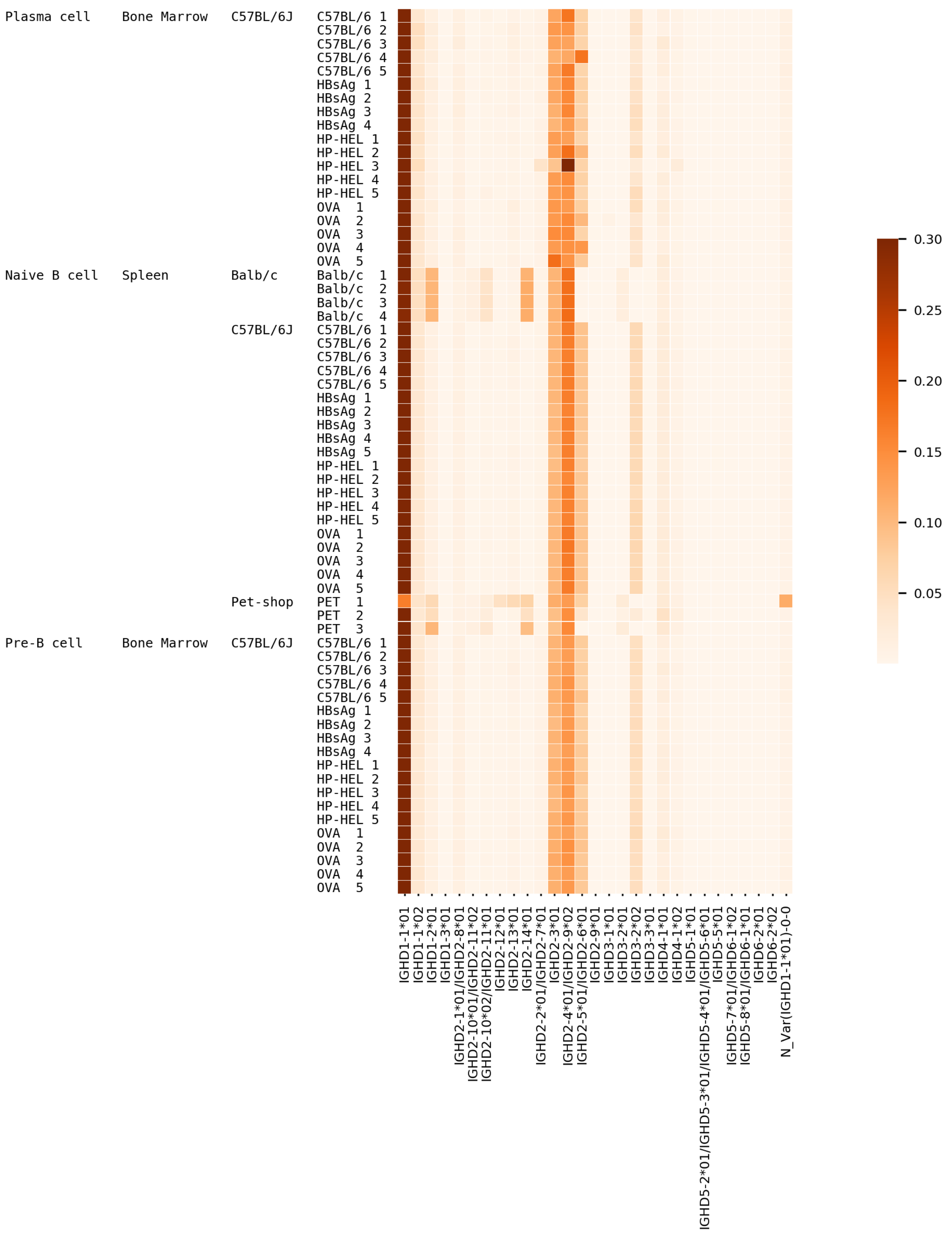

Figure M. Usage of various known and novel genes/variations in different datasets corresponding to different strain, cell type, and tissue from mice. Columns on the left represent cell type, tissue, strain, and individual, respectively. OVA, HP-HEL, and HBsAg in the right most column represent the C57BL/6J mice immunized with OVA, HP-HEL, and HBsAg, respectively. For example, OVA 3 represents the C57BL/6J mouse number 3 that was immunized with OVA.

Usage of D genes in the Rhesus macaque datasets. 52.6% of CDR3s on average were traceable in each dataset. The usage of the IMGT genes and the validated novel genes and variants of known genes is shown in Figure N.

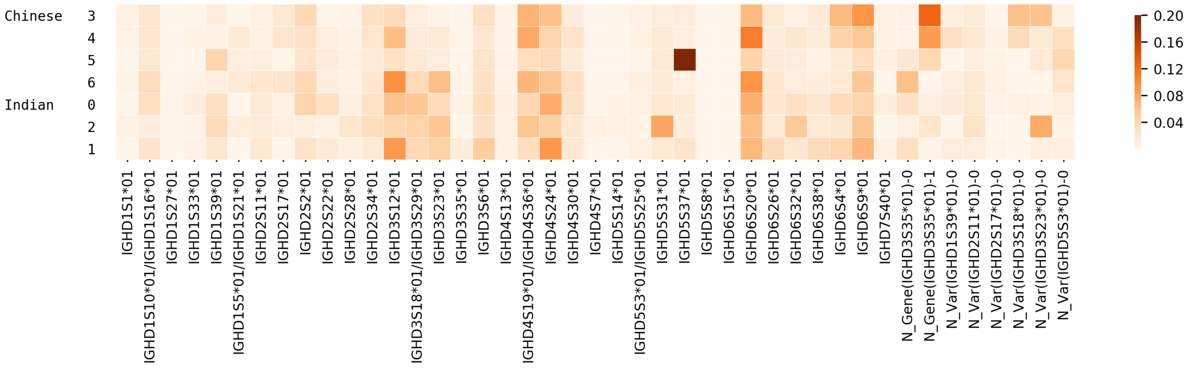

Figure N. Usage of known and novel genes in the Rhesus Macaque datasets. The novel genes and variations are shown on the right.

Usage of D genes in the Camel datasets. 31.7% of CDR3s on average were traceable in each dataset. Although the small sample size (n = 3) limits generalizability, the low number of traceable CDR3s could be due to high level of hypermutation within the CDR3 region as compared to other species. Since there is no IMGT database for camels, we used the alpaca IMGT database as a reference to analyze the usage. The usage of these genes and the validated novel variants of these genes is shown in Figure O. It can be seen that the D gene usage profiles are very different for the VH and the VHH isotypes within individuals, especially for individuals 2 and 3.

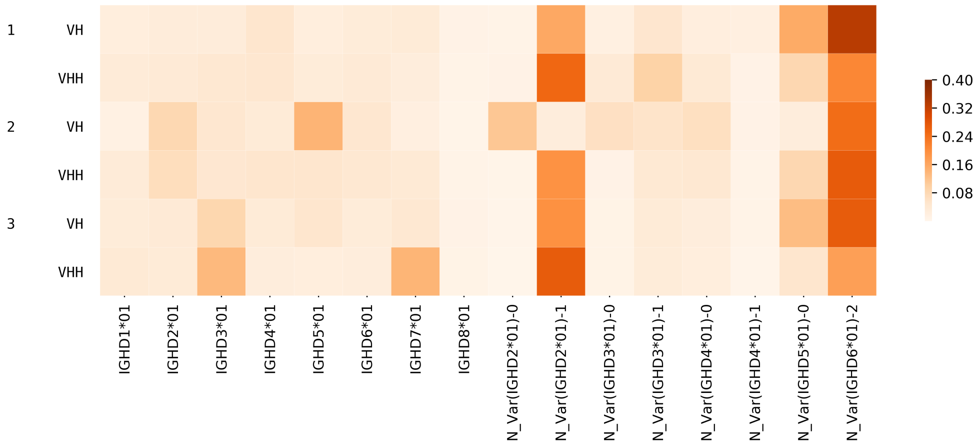

Figure O. Usage of known and novel genes in the Camel datasets.

Usage of D genes in the Rat datasets. 54.3% of CDR3s on average were traceable in each dataset. The usage of the IMGT genes and the validated novel variants is shown in Figure P. Genes belonging to the IGHD2 and IGHD3 families were underutilized as compared to other gene families, and the novel variants were among the genes that were utilized in most of the datasets. There is no clear distinction between the usage profiles between HuD and DNP immunized rats. This could be due to one or more of the following reasons: (a) the CDR3s here are from unsorted cells from spleen and not antigen specific cells; (b) the usage profiles of individuals might not be identical before immunization, hence masking the pattern if there was any.

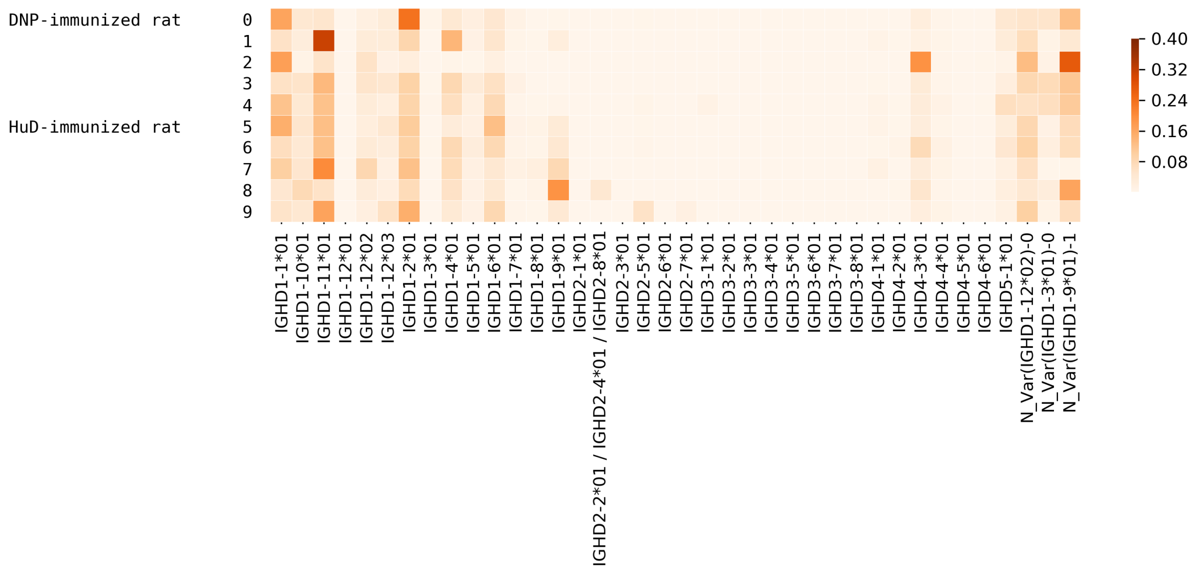

Figure P. Usage of D genes in the Rat datasets.

Supplemental Note: Overused D genes

Tables N-Q show overused genes in different datasets.

Gene	Donor	Tissue	Over-usage
IGHD1-7*01	M3	Brain Lesion	2.7x
		Cervical Lymph Node	3.1x
IGHD3-3*01	M4	Brain Lesion	2.6x
	M5	Brain Lesion	8.2x
		Choroid Plexus	4.7x

Table N. Overused genes in the Multiple Sclerosis datasets.

Gene	Donor	Tissue	Isotype	Cell type	Overusage
IGHD1-1*01	1	Colon	IgM	Memory B cell	2.0x
				Plasma cell	6.6x
IGHD2-21*01	0	Ileum	IgM	Memory B cell	5.0x
	3	Colon			3.6x
IGHD2-8*02	0	Ileum	IgA	Memory B cell	2.7x
				Plasma cell	2.2x
			IgM	Memory B cell	2.0x
IGHD3-16*02	0	Colon	IgM	Memory B cell	5.4x
				Plasma cell	3.3x
IGHD4-23*01	1	Colon	IgA	Plasma cell	2.3x
			IgM	Memory B cell	2.3x
IGHD6-25*01	0	Colon	IgM	Plasma cell	2.4x
		Ileum		Plasma cell	5.4x
				Memory B cell	2.0x
	3	Colon	IgA	Memory B cell	5.2x
				Plasma cell	3.1x

Table O. Overused genes in the Intestinal Repertoire datasets.

Gene	Isotype	Individual	Cell type	Over usage
IGHD1-1*01	IgG	16	HLA-DR+ Plasma cells	3.5x
			Total B cells	2.3x
IGHD3-10*02		14	HLA-DR+ Plasma cells	43.8x
			Total B cells	13.3x
		5	HLA-DR+ Plasma cells	2.1x

		2		5.7x
		8	HBsAg+ B cells	2.1x
IGHD3-3*01		12	HLA-DR+ Plasma cells	2.4x
		1	HBsAg+ B cells	2.9x
		8	HLA-DR+ Plasma cells	2.0x
IGHD6-25*01		13	HLA-DR+ Plasma cells	2.2x
		16		2.4x
		2		4.8x
		8	HBsAg+ B cells	2.4x
		2		2.6x

Table P. Overused genes in the Hepatitis B vaccination datasets

Gene	Individual	Overusage
IGHD7-27*01	CB1	3.4x
	CB2	3.8x
	CB3	3.8x
	CB4	4.6x

Table Q. Overused genes in the Cord Blood datasets.

Supplemental Note: Highly Used D Genes in Non-human Datasets

To find the genes with the highest usage among the datasets of a species, we picked the top 3 genes from each dataset. A gene is said to be *highly used* in all datasets from a species if it is one of the top 3 genes in at least 3 datasets. We found 3 highly used D genes for camels, 3 for macaques, and 4 for rats (Table R and Figure Q).

**Species (Total datasets)**	**D Gene**	**Datasets**
Camel (6)	N_Var (IGHD2*01)-1<br>N_Var (IGHD5*01)-0<br>N_Var (IGHD6*01)-2	5<br>3<br>6
Macaques (7)	IGHD3S12*01<br>IGHD4S19*01/IGHD4S36*01<br>IGHD6S20*01	3<br>3<br>5
Rats (10)	IGHD1-1*01<br>IGHD1-11*01<br>IGHD1-2*01<br>N_Var (IGHD1-9*01)-1	5<br>7<br>6<br>5

Table R. Highly used D genes in the Camel, Macaque, and Rat datasets. Genes shown here are among the top 3 genes in terms of usage proportion in the number of datasets shown in the right column.

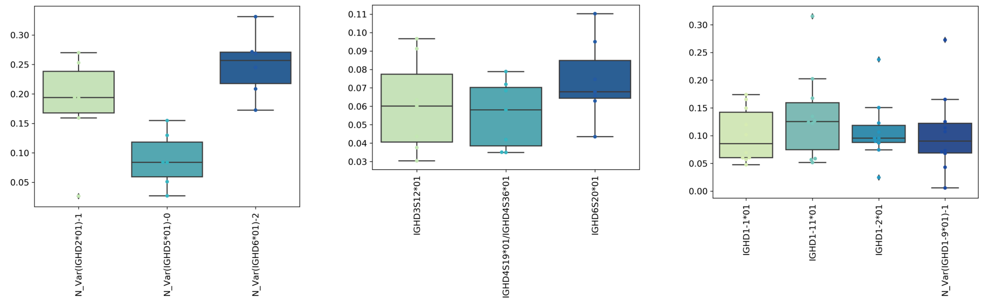

Figure Q. Usage proportion of highly used genes in the Camel (left), Macaque (middle), and Rat (right) datasets.

Supplemental Note: Benchmarking MINING-D on simulated CDR3s

We simulated 250,000 mutation-free CDR3s using the human D genes listed in the IMGT database (except for IGHD1-14*01, IGHD4-23*01, IGHD5-24*01) and IgSimulator tool [6]. We then generated four mutated versions of each of these CDR3s using mutation rates equal to 0.01, 0.05, 0.1, and 0.2. In total, we had one unmutated and four mutated datasets resulting in the average number of SHMs per CDR3 equal to 0, 0.7, 3.7, 7.4, and 14.8, respectively. For datasets with mutability < 0.1, MINING-D inferred all genes except for IGHD7-27*01 and one of the allelic variants of the gene IGHD2-2. There were no missing or additional nucleotide bases in the inferred D genes as compared to the D genes used for simulating the CDR3s. The missed gene IGHD7-27*01 is the shortest human D gene (11 nucleotides) that cannot be inferred using the default value of k (k=10) for MINING-D. MINING-D inferred only one of the allelic variants IGHD2-2*01 and IGHD2-2*03 since they differ only at the first base as shown below.

```
IGHD2-2*01       AGGATATTGTAGTAGTACCAGCTGCTATGCC
IGHD2-2*02       AGGATATTGTAGTAGTACCAGCTGCTATACC
IGHD2-2*03       TGGATATTGTAGTAGTACCAGCTGCTATGCC
```

In the dataset with the mutation rate 0.1, in addition to missing the gene IGHD7-27*01, MINING-D inferred only one sequence for the three allelic variants of IGHD2-2 shown above. As the mutation rate increased to 0.2, a similar pattern was observed for IGHD3-16. Moreover, only the first sequence was inferred for genes IGHD1-20*01 and IGHD1-7*01 shown below.

```
IGHD1-20*01      GGTATAACTGGAACGAC
IGHD1-7*01       GGTATAACTGGAACTAC
```

For the dataset with the mutation rate 0.2, multiple partial sequences were inferred per gene for some of the genes. However, there were no falsely inferred genes. For example, the following two sequences were inferred for the gene IGHD2-15*01

```
IGHD2-15*01      AGGATATTGTAGTGGTGGTAGCTGCTACTCC
Inferred-1         GATATTGTAGTGGTGGTAG
Inferred-2               GTAGTGGTGGTAGCTGCTAC
```

Mutation rate	Avg #SHMs per CDR3	#D genes used in the simulation	#D genes inferred by MINING-D	Missing D genes	Mean #missing/ extra nucleotides per inferred gene	Falsely inferred genes
0	0	29	27	IGHD2-2*03<br>IGHD7-27*01	0	0
0.01	0.74	29	27	IGHD2-2*01<br>IGHD7-27*01	0	0
0.05	3.70	29	28	IGHD2-2*01<br>IGHD7-27*01	0	1
0.1	7.40	29	26	IGHD7-27*01<br>IGHD2-2***	1.04	0
0.2	14.79	29	33	IGHD7-27*01<br>IGHD2-2***<br>IGHD3-16***	8.21	0

				IGHD1-20/1-7***		

Table S. Results of MINING-D on simulated datasets. X*** denotes that only a single allelic variant sequence was inferred among multiple allelic variants of a D gene X. X/Y*** denotes that only a single sequence was inferred for two D genes X and Y.

Supplemental Note: Benchmarking MINING-D on TCR datasets

We downloaded ten TRB cell datasets corresponding to 7 individuals from the immuneACCESS database (Table T). Each dataset consists of short sequences (~100 nt) fully covering CDR3s and partially covering V and J genes. Since our preprocessing step is not designed for such short sequences, we skipped CDR3 search step and used original sequences as an input for MINING-D. Information about the D genes in the TRBD locus and the datasets they were inferred from is provided in Table U. MINING-D inferred 2 genes in most of the datasets, however in 3 datasets, 3 genes were inferred. The falsely inferred genes (shown in Table V) are substrings of TRBV genes and are inferred because the input sequences partially cover V genes.

#	Dataset	immuneACCESS sample name	# productive rearrangements	Description	# Inferred D genes
1	Subj1_1	Subject1_Tcells_aliquot24	117,292	Control, Sorted Cells, Subject 01, T cells, deep, gDNA, site 07	2
2	Subj1_2	Subject1_Tcells_aliquot26	132,807	Control, Sorted Cells, Subject 01, T cells, deep, gDNA, site 07	3
3	Subj2_1	Subject2_Tcells_aliquot24	112,172	Control, Sorted Cells, Subject 02, T cells, deep, gDNA, site 07	2
4	Subj2_2	Subject2_Tcells_aliquot26	130,789	Control, Sorted Cells, Subject 02, T cells, deep, gDNA, site 07	3
5	Subj3_1	Subject3_PBMC_aliquot24	83,347	Control, PBMC, Peripheral blood lymphocytes (PBL), Subject 03, T cells, deep, gDNA, site 07	2
6	Subj3_2	Subject3_PBMC_aliquot26	110,776	Control, PBMC, Peripheral blood lymphocytes (PBL), Subject 03, T cells, deep, gDNA, site 07	2
7	BM4385_1_TCRB	BM4385_1_TCRB	27,965	151-180 lbs, 18-35 Years, 5ft 11in - 6ft 2in, Bone marrow, HIV Neg, Hepatitis B Virus Negative, Hepatitis C Virus Negative, Hispanic, Hispanic Ethnicity, O	3
8	BM4359_1_TCRB	BM4359_1_TCRB	87,283	121-150 lbs, 18-35 Years, 5ft 6in - 5ft 10in, Bone marrow, HIV Neg, Hepatitis B Virus Negative, Hepatitis C Virus Negative, Hispanic, Hispanic Ethnicity	2
9	BM4359_1_TCRB	BM4359_1_TCRB	59,563	151-180 lbs, 18-35 Years, 5ft 6in - 5ft 10in, African American Ethnicity, African Race, B, Bone marrow, HIV Neg, Hepatitis B Virus Negative, Hepatitis C Virus Negative	2

10	BM4359_1_TCRB	BM4359_1_TCRB	36,703	18-35 Years, 181-210 lbs, 5ft 6in - 5ft 10in, A, Bone marrow, Caucasian, HIV Neg, Hepatitis B Virus Negative, Hepatitis C Virus Negative	2

Table T. Description of human TRB datasets. Datasets 1–6 belong to the "TCRB technical replicates of PBMC from four donors" project. Datasets 7–10 belong to the "Bone Marrow From Healthy Adults" project. Both projects are available at the immuneACCESS database by Adaptive Biotechnologies. For the "TCRB technical replicates of PBMC from four donors" project, we did not use datasets corresponding to the donor 4 because they are too small. The numbers of productive rearrangements and descriptions were taken from the immuneACCESS database.

Gene	Variant	Sequence	Datasets inferred in	Datasets NOT inferred in
TRBD1	TRBD1*01	GGGACAGGGGGC	–	BM4384
TRBD2	TRBD2*01	GGGACTAGCGGGGGGG	BM4374<br>BM4385	–
	TRBD2*02	GGGACTAGCGGGAGGG	–	BM4374<br>Bm4385

Table U. Information about inferred D genes from TCR datasets using MINING-D.

Sequence	Datasets inferred in	Comments
TGTATCTCTGTGCCACC	Subj2_2<br>BM4384	substring of TRBV23/OR9-2*01
TCTGTGCCAGCAGTTAC	Subj1_2	substring of TRBV6-2/TRBV6-3/TRBV6-5/TRBV6-6
TGTACTTCTGTGCCA	BM4385	substring of TRBV6-2/TRBV6-3/TRBV6-5/TRBV6-6

Table V. Information about falsely inferred D genes from TCR datasets using MINING-D. All three sequences are substrings of some V genes.

Supplemental Note: Non-genomic insertions in naive and cord blood Rep-Seq datasets

We compared V-D and D-J insertions in datasets extracted from cord blood and naive B cells (Figure R). The median length of V-D insertions is 5 and 4 nt in naive and cord blood datasets, respectively. The difference between the V-D insertion lengths in naive and cord blood datasets is statistically significant, p-value is 4.14e-25 (according to the Kruskal-Wallis test). The median length of D-J insertions is 3 and 0 nt in naive and cord blood datasets, respectively. The difference between the D-J insertion lengths in naive and cord blood datasets is statistically significant, p-value is 5.61e-251. Thus, we confirm that B cells from cord blood have shortened (or even missing) insertions compared to B cells from naive datasets.

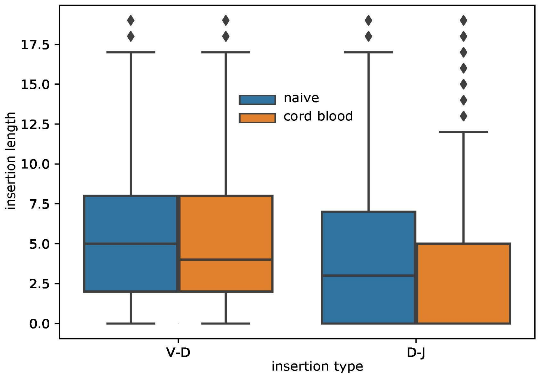

Figure R. Lengths of V-D and D-J insertions in naive (blue) and cord blood (orange) datasets. For the comparison, we used 3 naive datasets from the PRJNA355402 project (accession numbers are SRR5063084, SRR5063092, and SRR5063097) and 4 cord blood datasets from the PRJNA393446 project (SRR5811753, SRR5811754, SRR5811755, and SRR5811756). For better visualization, we discarded several long insertions from naive datasets (with lengths exceeding 20 nt).

References

1. Gusfield D. Algorithms on strings, trees, and sequences : computer science and computational biology. Cambridge England ; New York: Cambridge University Press; 1997. xviii, 534 pages p.
2. Souto-Carneiro MM, Sims GP, Girschik H, Lee J, Lipsky PE. Developmental changes in the human heavy chain CDR3. J Immunol. 2005;175(11):7425-36. doi: 10.4049/jimmunol.175.11.7425. PubMed PMID: 16301650.
3. Briney BS, Willis JR, Hicar MD, Thomas JW, Crowe JE. Frequency and genetic characterization of V(DD)J recombinants in the human peripheral blood antibody repertoire. Immunology. 2012;137(1):56-64. doi: 10.1111/j.1365-2567.2012.03605.x. PubMed PMID: 22612413; PubMed Central PMCID: PMCPMC3449247.
4. Elhanati Y, Sethna Z, Marcou Q, Callan CG, Mora T, Walczak AM. Inferring processes underlying B-cell repertoire diversity. Philos Trans R Soc Lond B Biol Sci. 2015;370(1676). doi: 10.1098/rstb.2014.0243. PubMed PMID: 26194757; PubMed Central PMCID: PMCPMC4528420.
5. Kidd MJ, Jackson KJ, Boyd SD, Collins AM. DJ Pairing during VDJ Recombination Shows Positional Biases That Vary among Individuals with Differing IGHD Locus Immunogenotypes. J Immunol. 2016;196(3):1158-64. Epub 2015/12/23. doi: 10.4049/jimmunol.1501401. PubMed PMID: 26700767; PubMed Central PMCID: PMCPMC4724508.
6. Safonova Y, Lapidus A, Lill J. IgSimulator: a versatile immunosequencing simulator. Bioinformatics. 2015;31(19):3213-5. Epub 2015/05/25. doi: 10.1093/bioinformatics/btv326. PubMed PMID: 26007226.
